# Cytosine methylation of mature microRNAs inhibits their functions and is associated with poor prognosis in glioblastoma multiforme

**DOI:** 10.1186/s12943-020-01155-z

**Published:** 2020-02-25

**Authors:** Mathilde Cheray, Amandine Etcheverry, Camille Jacques, Romain Pacaud, Gwenola Bougras-Cartron, Marc Aubry, Florent Denoual, Pierre Peterlongo, Arulraj Nadaradjane, Joséphine Briand, Farida Akcha, Dominique Heymann, François M. Vallette, Jean Mosser, Benjamin Ory, Pierre-François Cartron

**Affiliations:** 1 CRCINA, INSERM, Université de Nantes, Nantes, France; 2 Faculté de Médecine, Université de Nantes, Nantes, France; 3 Present address: Department of Oncology-Pathology, Cancer Centrum Karolinska (CCK), R8:03, Karolinska Institutet, SE-171 76 Stockholm, Sweden; 4 CNRS, UMR 6290, Institut de Génétique et Développement de Rennes (IGdR), F-35043 Rennes, France; 5 Université Rennes1, UEB, UMS 3480 Biosit, Faculté de Médecine, F-35043 Rennes, France; 6 Plate-forme Génomique Environnementale et Humaine Biosit, Université Rennes1, F-35043 Rennes, France; 7 CHU Rennes, Service de Génétique Moléculaire et Génomique, F-35033 Rennes, France; 8 INSERM, UMR 1238, équipe labellisée ligue 2012, 1 Rue Gaston Veil, 44035 Nantes, France; 9 LaBCT, Institut de Cancérologie de l’Ouest, Saint Herblain, France; 10 LabEx IGO “Immunotherapy, Graft, Oncology”, Nantes, France; 11 Cancéropole Grand-Ouest, réseau Epigénétique (RepiCGO), Nantes, France; 12 EpiSAVMEN, Epigenetic consortium Pays de la Loire, Nantes, France; 13 IRISA Inria Rennes Bretagne Atlantique, équipe GenScale, Campus de Beaulieu, 35042 Rennes, France; 14 Ifremer, Laboratoire d’Ecotoxicologie, Rue de l’Ile d’Yeu, BP21105, cedex 03 44311 . Nantes, France; 15 Institut de Cancérologie de l’Ouest, CRCINA INSERM U1232, Equipe 9 –Apoptose et Progression tumorale, LaBCT, Boulevard du Pr J Monod, 44805 Saint-Herblain, France

**Keywords:** miRNA, Cytosine-methylation, Epigenetics, DNMT3A, AGO4, Glioblastoma, Epitranscriptomics

## Abstract

**Background:**

Literature reports that mature microRNA (miRNA) can be methylated at adenosine, guanosine and cytosine. However, the molecular mechanisms involved in cytosine methylation of miRNAs have not yet been fully elucidated. Here we investigated the biological role and underlying mechanism of cytosine methylation in miRNAs in glioblastoma multiforme (GBM).

**Methods:**

RNA immunoprecipitation with the anti-5methylcytosine (5mC) antibody followed by Array, ELISA, dot blot, incorporation of a radio-labelled methyl group in miRNA, and miRNA bisulfite sequencing were perfomred to detect the cytosine methylation in mature miRNA. Cross-Linking immunoprecipiation qPCR, transfection with methylation/unmethylated mimic miRNA, luciferase promoter reporter plasmid, Biotin-tagged 3’UTR/mRNA or miRNA experiments and in vivo assays were used to investigate the role of methylated miRNAs. Finally, the prognostic value of methylated miRNAs was analyzed in a cohorte of GBM pateints.

**Results:**

Our study reveals that a significant fraction of miRNAs contains 5mC. Cellular experiments show that DNMT3A/AGO4 methylated miRNAs at cytosine residues inhibit the formation of miRNA/mRNA duplex and leading to the loss of their repressive function towards gene expression. In vivo experiments show that cytosine-methylation of miRNA abolishes the tumor suppressor function of miRNA-181a-5p miRNA for example. Our study also reveals that cytosine-methylation of miRNA-181a-5p results is associated a poor prognosis in GBM patients.

**Conclusion:**

Together, our results indicate that the DNMT3A/AGO4-mediated cytosine methylation of miRNA negatively.

**Graphical abstract:**

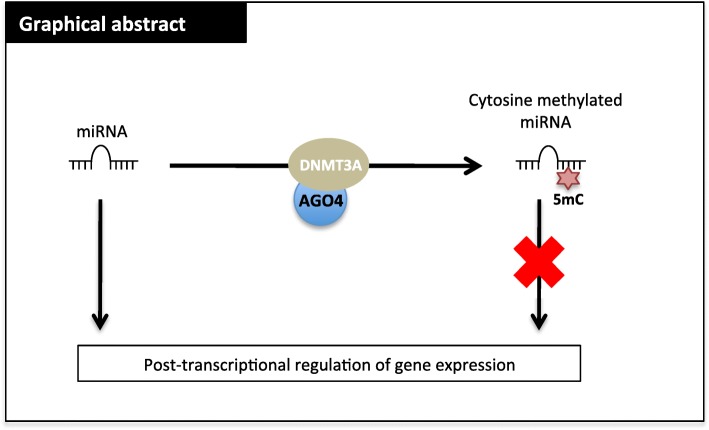

## Background

MicroRNAs (miRNAs) are short, single-stranded RNA molecules implicated in transcriptional and post-transcriptional regulation of gene expression [1, 2]. miRNAs target the RNA interference effector complex (RISC) of specific messenger RNAs (mRNAs) through partial base paring of sequences found predominantly in the 3’ untranslated region (UTR) of the gene. This reaction in turn increases the degradation of the mRNA and/or decreases its translation [3]. miRNAs have emerged as the key regulators in a wide variety of biological processes, including cell growth, proliferation and survival [4]. As a consequence, given the importance of their functions, miRNAs can act as both oncogenes (oncomiR) or as tumor suppressors, and it has been shown that they play crucial roles in the initiation, maintenance, and progression of oncogenesis in numerous types of cancer [5]. Several studies have shown that epigenetic changes in the promoter or coding region of miRNAs regulate their expression and thus the whole gene expression profile [6, 7]. In addition to this regulation, miRNAs can also be regulation via mechanims of base or phosphate modifications. Xhelmace et al. (2012) reported that BCDIN3D phospho-dimethylates pre-miRNA-145 both in vitro and in vivo and that phospho-dimethylated pre-miRNA-145 displays reduced processing by Dicer in vitro [8]. Alcarcón et al. (2015) reported that primary-miRNA can be adenosine-methylated and that hat this methylation acts as a key post-transcriptional modification that promotes the initiation of miRNA biogenesis [9]. Berulava et al. (2015) reported the presence of N6-adenosine methylation in miRNAs, and that his base modification affected the biogenesis and/or the stability of miRNAs [10]. Ma et al. (2017) reported that adenosine methylation of miRNAs positively modulates the primary miRNA process [11]. At molecular level, METTL3 (Methyltransferase-like 3) [9], WTAP (Wilms tumor 1-associated protein) [12] and METTL14 [11] were identified as key actors of adenosine methylation of miRNAs, and FTO (Fat mass and obesity-associated protein) [10] was identified as a key actor of adenosine demethylation of miRNAs. Recently, two other papers have been published on base or phosphate modifications of miRNAs. Pandolfini et al. (2019) reported that miRNA can be guanosine-methylated by METTL1, and that this methylation promoted miRNA processing [13]. Konno et al. (2019) confirmed that miRNAs could be adenosylmethylated and showed that miRNAs could be cytosine-methylated [14]. Konno et al. (2019) also reported that adenosine and cytosine methylated miRNA-17-5p can be used as biomarker of early-stage pancreatic cancer [14]. Despite, the undoubtable importance of this first report concerning the cytosine methylation of miRNAs, many scientific questions concerning cytosine methylation remaind unanswered: how cytosine methylation of miRNAs impacts their functionality? What is the molecular player of miRNAs cytosine methylation?.

Over several years, we have started research programs aimed at i) determining the putative presence of 5-methylcytosine in miRNAs, ii) identifying the molecular actors of this methylation, iii) to investiagte the impact of the cytsone methylation of miRNAs on their functionality and iv) determining whether cytsoine-methylated miRNAs can be used as biomarker in the glioblastoma multiforme (GBM).

Our study show that miRNAs are cytosine-methylated in GBM cells and tumor samples. DNMT3A/AGO4 was identified as a player in miRNAs cytosine-methylation, and that this modification has a negative influence on miRNA-mediated gene regulation.

## Methods

### miRNA extraction

miRNA extractions were performed using the NucleoSpin® miRNA kit (Macherey Nagel, France) according to the manufacturer’s instructions. 5.10^6^ cultured cells or 15 mg of tissue were used for one extraction. Purification of isolated miRNA was then investigated in 5% agarose gel electrophoresis. Image acquisition was performed on ChemiDoc MP (Bio-Rad, France).

### RNA-immunoprecipitation for miRNA

RIP (RNA-ChIP) was performed as described previously [15]. All buffers used in this study contained 0.5 U/μL RNase inhibitor. First, the nuclei from the cells were isolated from 1% formaldehyde-fixed cells and used for chromatin fragmentation. After immunoprecipitation with the antibodies of interest, the beads were washed, then the RNA was eluted and precipitated with ethanol. The precipitated RNA pellets were resuspended in nuclease-free water containing RNase inhibitor, 50 mM Tris-HCl, pH 7.5, 10 mM MgCl2, and DNase I. The mixture was incubated for 30 min at 37 °C and extracted once with phenol/chloroform. The RNA was then precipitated with ethanol and dissolved in nuclease-free water. An aliquot of RNA was used for a cDNA synthesis reaction and qPCR analysis. Fold enrichment was next calculated using Ct value obtained from miScript miRNA PCR Array performed with input miRNA, IP-IgG and IP-m6A and the 2^-ΔΔCt^ formula.

### Quantitative PCR of miRNA

For miRNA expression analysis, the RNA was reverse transcribed using a miRScript II RT kit and analyzed by qPCR with the miScript SYBR Green PCR Kit using the specific hsa-miR miScript Primer Assays (Qiagen, France) according to the manufacturers’ instructions. miRs expression fold changes were calculated using the ^2-ΔΔCt^ formula and SNORD61 as normalizer according to the manufacturer’s instructions.

### RNA bisulfite sequencing of miRNA

Methylation of microRNAs was analyzed on both bisulfite-converted and non-converted (control condition) RNA samples. Libraries from three biological replicates were prepared from 30 ng of small RNAs with the NEBNext Small RNA Library kit (Biolabs, France), according to the manufacturer’s instructions. To correct the unbalanced base composition of the libraries prepared from converted small RNAs, we used a PhiX spike-in (10%). The libraries were sequenced on an Illumina MiSeq with Rapid SBS kit (50 cycles) (Illumina, France). Adapter sequences were removed using Cutadapt. Only reads with a sequence length of more than 16 bp were selected for further analysis and filtered according to their quality (Q Score ≥ 30). Reads of poor informational content were discarded; these included reads presenting a homopolymer sequence of more than 12 bases and reads with undetermined (N) terminal bases. Unique reads were counted and gathered under a single identifier in fasta format. Reads obtained from the sequencing of the non-converted RNA sample and the converted RNA samples were aligned on the pre-miRNA hairpin sequences downloaded from miRBase 21. Alignments were performed using an ad hoc python script designed for the mapping of RNA sequences on RNA references. When mapping converted RNA sequences, it has the particularity of authorizing mismatches between a nucleotide ‘T’ from a read, with a nucleotide ‘C’ from the bank. Otherwise, this script is a classical seed-and-extend heuristic. The ‘T’-'C’ mismatches are authorized during both the seed indexation phase and the extension phase. This tool is available under the GNU affero general public license from here: https://github.com/pierrepeterlongo/MethMap.git. The parameters were set in order to conserve reads with alignment (i) whose length equalled at least 90% of the read length, (ii) at least 80% of the targeted mature miRNA was covered by the alignment, and (iii) with no mismatches (except the authorized ‘T’-'C’ mismatches with converted reads). Non-uniquely mapped reads were assigned to the pre-miRNA on which they had the longest alignment. In case of equality, all targeted pre-miRNA were conserved. For each mature miRNA expressed in the converted RNA sample, we calculated a percentage of methylation as the ratio between the number of reads displaying a non-converted CG and the total number of reads mapped to the pre-miRNA sequence. For each pre-miRNA, we evaluated the bisulfite conversion rate as the ratio between the number of unconverted non-CpG cytosines (CpA, CpT, and CpC) and the total number of non-CpG cytosines covered by at least one read. We discarded the pre-miRNA (i) not expressed in the control sample, (ii) covered by less than 25 reads and, (iii) with an estimated conversion rate of less than 95% (Additional_file_pipeline.pdf).

### Biotin-tagged miRNA or 3’UTR/mRNA experiments

These experimentations were carried out as previously described [16]. Synthetic biotin-labeled miRNA duplexes (200 pmoles) were transfected into 4.10^6^ cells using HiPerFect Transfection Reagent (Qiagen, France). Cells were harvested after 24 h, and lysed in hypotonic lysis buffer (10 mM KCl, 1.5 mM MgCl2, 10 mM Tris-Cl pH 7.5, 5 mM DTT, 0.5% NP-40, 60 U/ML SUPERase) and 1× Complete Mini protease inhibitor (Roche, France). Cell debris was cleared by centrifugation (≥ 10,000 g at 4 °C for 2 min). The supernatant was transferred to a clean tube, and NaCl was added to a final concentration of 1 M. Dynabeads (25 μl; Thermo, France) were pre-blocked with 1 μg/μl bovine serum albumin and 1 μg/μl yeast tRNA (Thermo, France), and incubated with the supernatant for 30 min at room temperature. Beads were then washed with hypotonic lysis buffer and 1 M NaCl before RNA or miRNA extraction using an appropriate kit (Qiagen) and according to the manufacturer’s instructions. The qPCR calculations take into consideration the Ct obtained from qPCR performed with miRNA or mRNA extract (as input), 3’UTR/BIM or miRNA-181a-5p and mutated 3’UTR/BIM or unspecific miRNA (as negative control) and the 2^-ΔΔCt^ method.

## Results

### 5-methylcytosine marks are found in mature miRNA

**Fig. 1:**
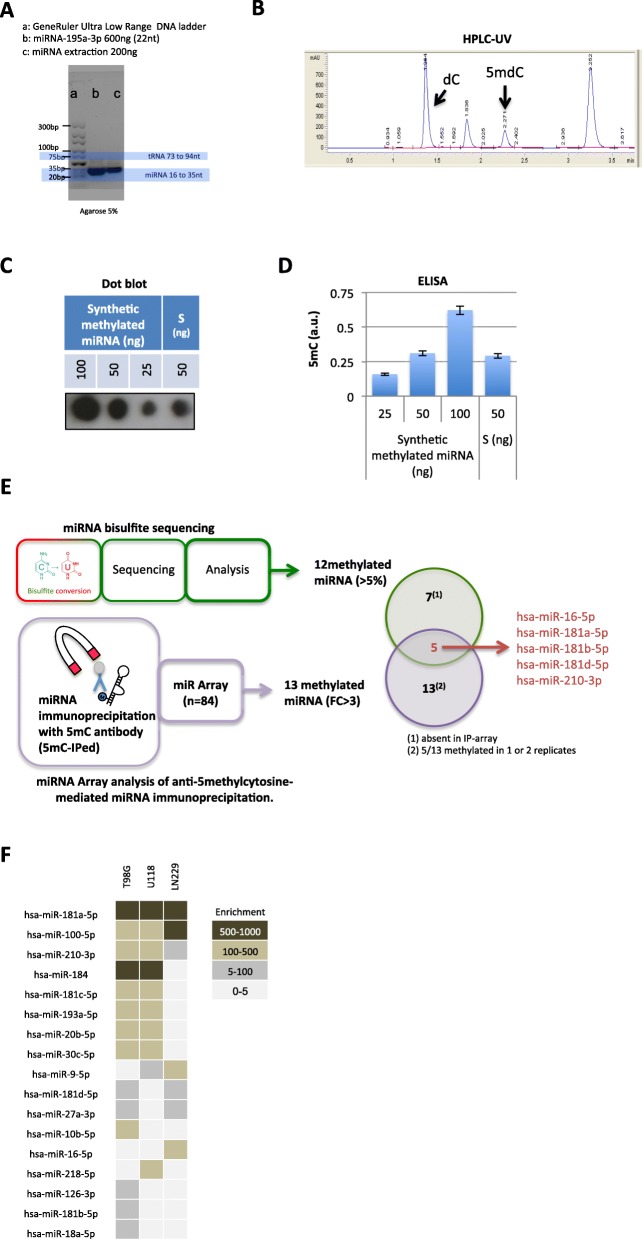
Five different methods detect the presence of 5-methycytosine in miRNA. **a** Picture illustrates miRNA migration in 5% agarose gel electrophoresis. Image acquisition was performed on ChemiDoc MP (Bio-Rad, France). **b** HPLC chromatograms of 5-methylcytosine (5mC) in miRNA. According to the calibration with standard dC and 5mdC, peaks with a retention time of 1.364 and 2.271 were attributed to dC and 5mdC, respectively. **c** Picture illustrates a Dot blot using 5mC antibody. miRNA were spotted on to a positive-charged nylon membrane and blotted with 5mC antibody (Active Motif, France). “S” sample of miRNA extracted to glioblastoma cells. In an arbitrary manner, we decided to use the synthetic methylated miRNA-4665-3p as control as this miRNA was composed of 6 CG (MIMAT0019740 # CUCGGCCGCGGCGCGUAGCCCCCGCC, according to the miRBase website.) Image acquisition was performed on ChemiDoc MP (Bio-Rad, France). **d** Graph illustrates the quantification of the miRNA sample using the ELISA method (Methylamp Global DNA Methylation Quantification kit, Epigentek-Euromedex, France). Data (averagestandard deviation) are representative of three independent experiments. **e** Schematic representation of miRNA bisulfite sequencing and miRNA Array analysis (Qiagen, France) of anti-5methylcytosine-mediated miRNA immunoprecipitation. **F.** Heatmap representation for adenosine methylated enrichment of miRNA in three gliobklastoma cell lines: T98G, U118 and LN229

Methylation of cytosine to form 5-methylcytosine (5mC) is a chemical modification commonly seen in DNA and thus, is a possibility in miRNAs. To verify this hypothesis, we analyzed the 5mC contents of miRNAs using 5 distinct methods. The analyses were performed after miRNA extraction from U87 cells, a glioblastoma cell line. Gel electrophoresis and an Agilent Small RNA kit (Agilent Bioanalyzer 2100) showed the integrity and purity of the extracted miRNA **(**Fig. 1a and Fig. S1a). RT-qPCR performed with tRNA-specific primers validated the absence of tRNA in our miRNA extraction (Fig. S1b). The presence of 5mC in miRNA was determined by HPLC-UV (Fig. 1b and Fig. S2), by dot blot (Fig. 1c) and ELISA (Fig. 1d).

Since the latter experiments were based on miRNA extraction that could be contaminated by small RNA (such as tRNA-derived fragments or piRNA), we cannot rule out that the 5mC signal detected in ELISA, HPLC and dot blot could be due to this contamination. To overcome this, two methods were used to identify miRNA sequences: bisulfite conversion followed by high-throughput sequencing analysis and adequate small RNAs databases (miRNA-BSeq) and 5mC immunoprecipitation followed by miRNA array analysis.

Figure S3 shows the miRNA-BSeq workflow. A total of 2565 unique mature miRNA sequences were downloaded from miR-Base-21. Among them 892 (35%) had at least one CpG dinucleotide. Results obtained from three biological replicates (bisulfite-converted samples BS1, BS2, BS3 and corresponding non-converted control samples noBS1, noBS2, noBS3) are presented in table_sequencing_results.xls (Raw FASTQ data have been submitted to ArrayExpress). As previously described in the non-converted samples, the oncogenic miRNA-21-5p was over-expressed, and represented around 25% of the total mapped reads (data not shown). Under the converted conditions, sequenced reads mapped to 644, 649, and 659 miRNAs in BS1, BS2, and BS3 conditions, respectively (additional_file_all_mir.xls). After expression level filtering, we selected 114, 122, and 118 miRNAs in BS1, BS2, and BS3 conditions respectively. These three selections largely overlapped as 102 miRNAs were identified in all replicates. We focused on the methylation levels of CpG dinucleotides for 22 (BS1), 28 (BS2), and 23 (BS3) miRNAs displaying a conversion rate higher than 95% (additional_file_selected_mir_BSx.zip). Methylation levels strongly correlated for 17 miRNAs present in the three replicates (Pearson correlation coefficients ranged from 0.7 to 0.8). The five miRNAs displaying the highest percentage of methylation were miRNA-16-5p (24%), hsa-miR-29c-3p (11%), hsa-miR-210-3p (9%), hsa-miRNA-181a-5p (9%), and hsa-miRNA-339-3p (9%) (additional_file_selected_mir.xls). These results were validated by miRNA Array technology on 5mC-immunoprecipitated miRNAs. Of the 12/17 methylated miRNAs (percentage of methylation ≥5), five (miRNA-16-5p, miRNA-181a-5p, miRNA-181b-5p, miRNA-181d-5p and miRNA-210-3p) were also found methylated in miRNA Array analysis (FC enrichment ≥3) (Fig. 1e).

In addition, the sequence of these 5 miR was not recognized as piRNA or tRF (tRNA-derived fragments) following the use of two adequate databases (piRBase / http://www.regulatoryrna.org/database/piRNA and tRF/ http://genome.bioch.virginia.edu/trfdb/ [17, 18]).

To determine whether the 5-cytosine-methylation of miRNA-16-5p, miRNA-181a-5p, miRNA-181b-5p, miRNA-181d-5p and miRNA-210-3p was restricted to the U87 cells, we analyzed the 5-cytosine methylation level of miRNA in 3 other glioblastoma cell lines: T98G, U118 and LN229 using the miRNA Array technology on 5mC-immunoprecipitated miRNAs. Figure 1f (additional_file_miR_Enrichment.xls) show that three (miRNA-181a-5p, miRNA-100-5p and miRNA-210-3p) were communly found highly methylated (enrichment> 5) in T98G, U118 and LN229 cells. As a control, we used miRNA-181a-5p as a « demonstrator » since this miRNA had the most cytosine methylated in our panel of 4 GBM cell lines.

### DNMT3A/AGO4 methylates miRNAs

**Fig. 2:**
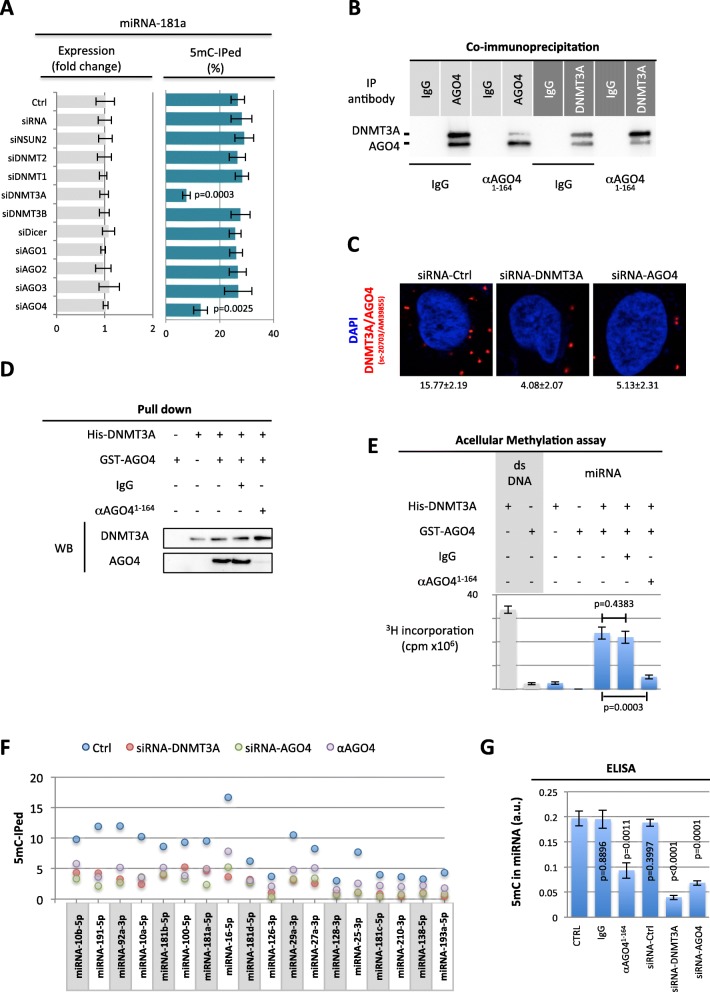
DNMT3A/AGO4 methylates miRNA. **a** siRNA against DNMT3A or AGO4 decreased the methylation level of miRNA-181a-5p. Expression of miRNA-181a-5p was assessed by qPCR and 5mC-IPed estimated the methylation level of the miRNA under the different siRNA tested. **b** Western blot experiments after immunoprecipitation using the Catch and Release® v2.0 Reversible Immunoprecipitation System (Milipore, France) and 4 μg IgG (negative control) or DNMT3A antibodies. Analysis of the DNMT3A or AGO4 expression after cell transfection with the indicated antibody. The Pro-Ject Protein Transfection Reagent kit (Thermo Scientific, France) was used to deliver antibodies in living cells according to the manufacturer’s instructions. IgG (10 μg) was used as a negative antibody control and α AGO4^1–164^, (10 μg, Active Motif (AM39855), France) an antibody directed against the 1–164 amino acid region of AGO4, was used as to block the DNMT3A/AGO4 interaction. **C.** Proximity Ligation In Situ Assays were performed to investigate the interaction or close proximity between DNMT3A and AGO4 in U87 cells treated with control siRNA, siRNA targeting DNMT3 or AGO4. Red dots represent DNMT3A/AGO4 interactions. Nuclei are stained with DAPI (blue). The quantification of DNMT3A/AGO4 interactions (average ± standard deviation) was performed in 30 cells in three independent experiments. **d** Western blot experiments were performed after His-pull-down assay using His-DNMT3A and GST-AGO4 as bait and prey proteins respectively. IgG (4 μg) was used as a negative antibody control and αAGO4^1–164^ (4 μg, Active Motif (AM39855), France), an antibody directed against the 1–164 amino acid region of AGO4, was used as blocker of DNMT3A/AGO4 interaction. **e** DNMT Magnetic Beads (DMB) Assay using DNMT3A and/or AGO4 (300 nM), AdoMet (900 nM), synthetic double-stranded DNA oligonucleotides (ds DNA) or synthetic miRNA. The mean values of triplicate experiments are presented with standard deviation error bars. IgG (4 μg) was used as a negative antibody control and αAGO4^1–164^ (4 μg, Active Motif (AM39855), France) to block the DNMT3A/AGO4 interaction. **f** Cytosine-methylation profile of miRNAs immunoprecipitated by an anti-5methylcytosine. The graph illustrates the cytosine-methylation level of the 18 miRNA identified as methylated via the miRIP-5mC/Array method (according to Fig. 1e) in U87 cells treated or not (blue circle) with siRNA-DNMT3A (red circle), siRNA-AGO4 (green circle) and αAGO4^1–164^ (purple circle). **g** 5mC quantification using ELISA in 100 ng of miRNA from cells treated or not with the indicated antibodies. The Pro-Ject Protein Transfection Reagent kit (Thermo Scientific, France) was used to deliver antibodies to living cells according to the manufacturer’s instructions. IgG (10 μg) was used as a negative antibody control and αAGO4^1–164^ (10 μg, Active Motif (AM39855), France) was used to block the DNMT3A/AGO4 interaction. Mean values of triplicate experiments presented with standard deviation error bars

To better characterize the biological process associated with the cytosine methylation of miRNA, we used an siRNA-mediated invalidation of DNA and RNA methyltransferases (e.g. DNMT1, 3a, 3b), as well as the proteins involved in miRNA biogenesis and the effector component of the miRNA-induced silencing complex (namely AGO 1–4) (Figure S4). We used miRNA-181a-5p as a read out for siRNA efficacy in methylation inhibition. 5mC-mediated co-immunoprecipitation (co-IP) of miRNA indicated that only siRNA-DNMT3A and siRNA-AGO4 decreased the methylation level of miRNA-181a-5p without changing its expression level (Fig. 2a). The latter result suggests that AGO4 and DNMT3A could form a complex responsible for the cytosine-methylation of miRNA-181a-5p, which raises two questions: do DNMT3A and AGO4 co-exist in the same complex; and if yes, does the DNMT3A/AGO4 complex promote miRNA cytosine-methylation in a general manner?

To determine whether DNMT3A and AGO4 were included in the same methylating complex, we first performed co-IP experiments. Figure 2b and Figure S5 show a co-IP between DNMT3A and AGO4. Intracellular transfection of an antibody raised against AGO4 (αAGO4^1–164^) specifically decreased the co-IP of Dnmt3A and AGO4, while the transfection of control IgG had no effect (Fig. 2b and Figure S5). We also observed with Proximity Ligation In Situ Assay (P-LISA, [19]) method the presence of an interaction between DNMT3A and AGO4 and, as expected, both siRNA decreased the DNMT3A/AGO4 interaction (Figs. 2c and Figure S6). Thirdly, cell-free pull-down experiments were performed to confirm the direct interaction between DNMT3A and AGO4 supposed by positive results of co-IP and P-LISA. In this assay, histidine-tagged DNMT3A was used as bait captured on an immobilized affinity ligand specific to the tag, and GST-labeled AGO4 was used as prey (materials and methods). As shown in Fig. 2d and Figure S7 the interaction between DNMT3A and AGO4 was confirmed and the αAGO4^1–164^ co-incubation decreased this interaction. This set of three distinct experiments support the idea that DNMT3A and AGO4 directly interact to form a complex.

To determine whether the DNMT3A/AGO4 complex promotes the cytosine-methylation of miRNA, we performed 3 different experiments. In an acellular system, we noted that the incorporation of radiolabeled methyl groups into the synthetic miRNA-181a-5p using DNMT3A was increased in the presence of AGO4 and that the addition of αAGO4^1–164^ decreased this incorporation (Fig. 2e). We then extended our study to a cellular system in which siRNA-DNMT3A, siRNA-AGO4 and αAGO4^1–164^ were used to decrease the integrity of the DNMT3A/AGO4 complex. We noted that all 3 additions decreased the cytosine-methylation of all the miRNAs identified as methylated using the miRIP-5mC/Array method (Fig. 2f and Figure S8). Under these conditions, a decrease in the global level of 5mC in miRNAs was observed in both ELISA and dot blot (Fig. 2g and Figure S9). This set of 3 distinct experiments support the idea that the DNMT3A/AGO4 complex is responsible for the cytosine-methylation of miRNA. This point is also enhanced by the positive correlation between the levels of cytosine-methylation of miRNA and DNMT3A/AGO4 interactions observed in a biological cohort of 32 GBM samples (Figure S10 and Supplementary Table T1).

### Cytosine-methylated miRNA-181a-5p loses its ability to interact with 3’UTR mRNA

As miRNAs play important roles in post-transcriptional gene regulation, we analyzed the effect of cytosine-methylation on this function. For this purpose, we decided to focus our research on the effect of miRNA-181a-5p on BIM since the regulation of apoptotic players are at the center of our research and BIM expression in GBM is crucial to calculate the BH3^score^, a biomarker associated with overall survival prognosis in GBM patients [20].

**Fig. 3:**
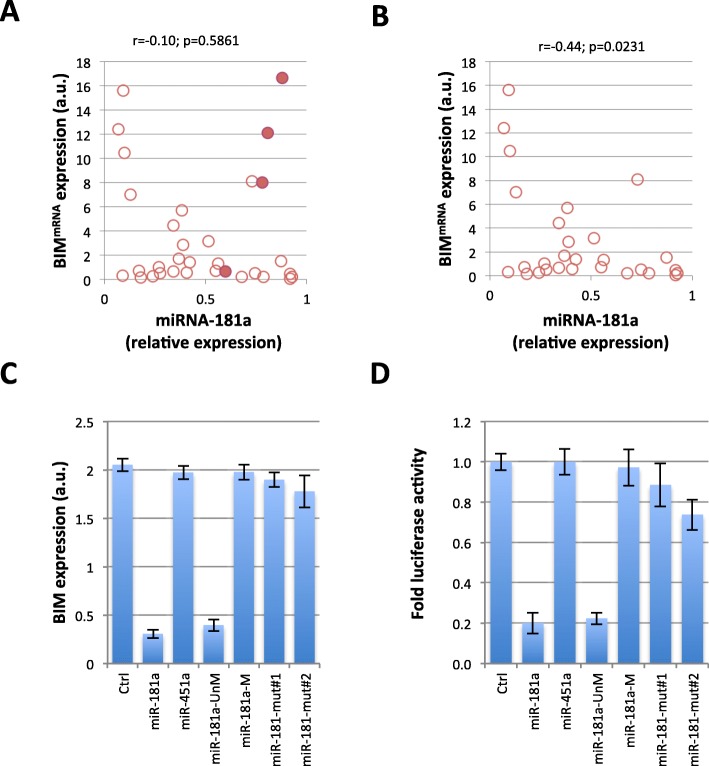
Cytosine-Methylated miRNA-181a-5p loses its repressor function. **a** Correlation study between miRNA-181a-5p and BIM protein expression determined in a cohort of 32 GBM samples. qPCR was used to determine the miRNA-181a-5p expression level. ELISA was performed to estimate BIM Expression. Each open circle represents a GBM sample. Pearson’s correlation test was used to measure the strength of the linear association between the two variables. **b** Correlation study between miRNA-181a-5p and BIM expression in the GBM samples in which miRNA-181a-5p was unmethylated. qPCR was used to determine the percentage of miRNA-181a-5p expression level. miRNA immunoprecipitation with 5mC antibody was performed to determine the methylation level of miRNA-181a-5*p. ELISA* was performed to estimate BIM expression. Each open circle represents a GBM sample. Pearson’s correlation test was used to measure the strength of the linear relationship between the two variables. **c** BIM expression level by ELISA in cells treated with indicated miRNAs. All miRNA (wild-type, mutated or methylated) were obtained from Sigma (France). **d** Impact of the methylation of miRNA-181a-5p on the BIM expression level via the 3’UTR interaction. Cells were transiently transfected with the indicated miRNA and a BIM 3’UTR-reporter or control reporter. Luciferase activity was determined 48 h after transfection

We first investigated the correlation between miRNA-181a-5p expression and one of its targets, the anti-apoptotic protein BIM (according to the miRTarBase) in our cohort of 32 GBM patient samples. ELISA was used to quantify BIM protein expression, qPCR were performed to quantify miRNA-181a-5p expression levels, and the 5mC level in miRNAs was determined by miRIP-5mC/qPCR. Figure 3a shows that there was no correlation between miRNA-181a-5p and BIM expression levels when all samples were considered. However, their expressions were correlated in samples in which miRNA-181a-5p was not methylated (Fig. 3b). Taking this into account we hypothesized that the presence of 5mC in miRNA-181a-5p abrogated its repression of BIM expression. This hypothesis was tested by treating U87 cells with either unmethylated, methylated or two forms of a mutated miRNA-181a-5p (Figure S11). One of these mutants (mut#1) was designed to lose its repressor function towards BIM in accordance with Taylor et al. (2013) [21], while the second mutant (mut#2: mutation of cytosine-10 and -16) was designed to loose its CG. miRNA-451a was used as a negative control as this miRNA does not target BIM. We observed a significant decrease in BIM expression in miRNA-181a-5p-transfected cells compared to the control (Fig. 3c and Supplemantary Figure S12). Interestingly, unmethylated miRNA-181a-5p reduced BIM expression, while the methylated and the two mutated miRNA-181a-5p did not affect BIM expression as compared to untreated cells or cells transfected with miRNA-451a.

To further investigate the role of miRNA-181a-5p on BIM regulation, the miRNA-181a-5p binding site on the BIM 3′-UTR was inserted into a 3′-UTR of a constitutively active luciferase reporter (pmiR-BIM-3’UTR). The luciferase activity of pmiR-BIM-3’UTR was significantly reduced by miRNA-181a-5p and unmethylated miRNA-181a-5p, but was not, or only weakly, affected in the methylated or with both mutated forms of miRNA-181a-5p (Fig. 3d).

Overall, our data demonstrate that the presence of 5mC on miRNA-181a-5p abolished its repressive function towards BIM. In addition, the mutation of cytosine-10 and -16 showed the same effect as the presence of 5mC on the function of miRNA-181a-5p towards BIM, suggesting that these two cytosines play a crucial role in the repressive function of miRNA-181a-5p.

### Cytosine-methylation of miRNA-181a-5p abolishes the formation of the miRNA-181a-5p-3’UTR/BIM duplex

**Fig. 4:**
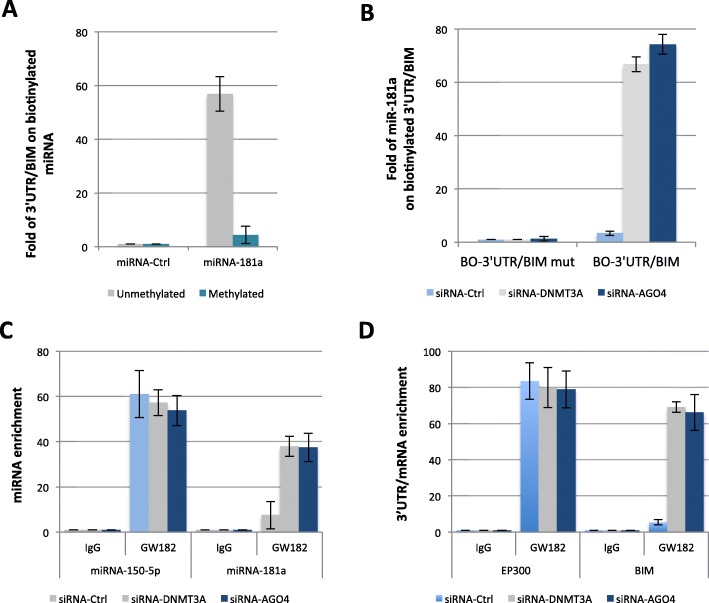
Cytosine-methylation of miRNA-181a-5p abolishes the formation of miRNA-181a-5p-3’UTR/BIM duplex. **a** The graph illustrates the relative presence of 3’UTR/BIM on biotinylated miRNA according to the previous method. **b** The graph illustrates the relative presence of miRNA-181a-5p on 3’UTR/BIM on biotinylated miRNA according to the previous method. **c** The graph illustrates the miRNA-150-5p and miRNA-181a-5p enrichments on GW182 and IgG (negative control). Experiments were performed using the RiboCluster Profiler kit (CliniScience, France) according to manufacturer’s instructions. **d** The graph illustrates the 3’UTR/BIM and 3’UTR/EP300 enrichments on GW182 and IgG (negative control). Experiments were performed using the RiboCluster Profiler kit (CliniScience, France) according to the manufacturer’s instructions

We then studied the formation of miRNA-mRNA duplex by performing biotin-tagged miRNA experiments [22, 23]. In these experiments, RT-qPCR quantified the amount of endogenous 3’UTR/BIM recruited on synthetic unmethylated or methylated biotin-tagged miRNA-181a-5p. Synthetic unmethylated or methylated biotin-tagged miRNA-1307 (mi-Ctrl) was used as a negative control. No amplification of 3’UTR/BIM was detected in either unmethylated or methylated biotin-tagged miRNA-1307 (Fig. 4a). 3’UTR/BIM amplification was detected in unmethylated and biotin-tagged miRNA-181a-5p, while no 3’UTR/BIM amplification was detected in methylated biotin-tagged miRNA-181a-5p (Fig. 4a). We thus concluded that the cytosine-methylation status of miRNA-181a-5p influenced duplex formation between endogenous 3’UTR/BIM and synthetic miRNA-181a-5p.

We then extended our experiments by using biotin-tagged 3’UTR/BIM. In these experiments, RT-qPCR quantified the amount of miRNA-181a-5p recruited to biotin-tagged 3’UTR/BIM. A mutated sequence of 3’UTR/BIM was used as negative control. To analyze the impact of the cytosine-methylation of miRNA-181a-5p on its recruitment to 3’UTR/BIM, biotin-tagged 3’UTR/BIM was transfected in cells with a siRNA-induced downregulated of DNMT3A or AGO4 (under these conditions a decrease in cytosine-methylation level of miRNA-181a-5p). We found that the binding of miRNA-181a-5p to the biotin-tagged 3’UTR/BIM had strongly increased in cells with DNMT3A or AGO4 invalidation (Fig. 4b). We thus concluded that the endogenous cytosine-methylation status of miRNA-181a-5p influenced duplex formation between synthetic 3’UTR/BIM and endogenous miRNA-181a-5p.

To reinforce the idea that the cytosine-methylation status of miRNA-181a-5p influences the endogenous formation of 3’UTR/BIM-miRNA-181a-5p duplex, we next performed Cross-Linking Immunoprecipitation and qPCR (CLIP-qPCR) analysis. In our assay, immunoprecipitation is performed using an antibody directed against GW182 (a protein of a RISC complex with a central role in miRNA-mediated silencing) and qPCRs were performed to detect the enrichment/presence of miRNA and 3’UTR/mRNA on the GW182-mediated co-immunoprecipitation products. CLIP-qPCR was performed on U87 cells invalidated for DNMT3A or AGO4 in order estimate the impact of the loss of cytosine-methylation on the GW182-mediated co-immunoprecipitation of miRNAs and mRNAs. The miRNA-150-5p-3’UTR/EP300 duplex was considered as control. The choice of this control was dictated by the fact that miRNA-150-5p is devoid of CpG and the fact that miRNA-150-5p targets 3’UTR/EP300 [24].

We noted that miRNA-150-5p and 3’UTR/EP300 were present in GW182-mediated co-immunoprecipitation products, and this independently of DNMT3A or AGO4 invalidation (Fig. 4c and Fig. 4d). Secondly, we noted that the DNMT3A or AGO4 invalidations strongly increased the presence of miRNA-181a-5p and 3’UTR/BIM on the GW182-mediated co-immunoprecipitation products (Fig. 4 c and d right). Thus, the latter results indicated that the cytosine-methylation status of miRNA-181a-5p influenced the endogenous formation of 3’UTR/BIM-miRNA-181a-5p-duplex.

In conclusion, CLIP-qPCR and biotin-tagged miRNA or 3’UTR/mRNA experimentations support the fact that cytosine-methylation of miRNA inhibits the formation of miRNA/mRNA duplex.

### Cytosine-methylation of miRNA-181a-5p modulates miRNA function

**Fig. 5:**
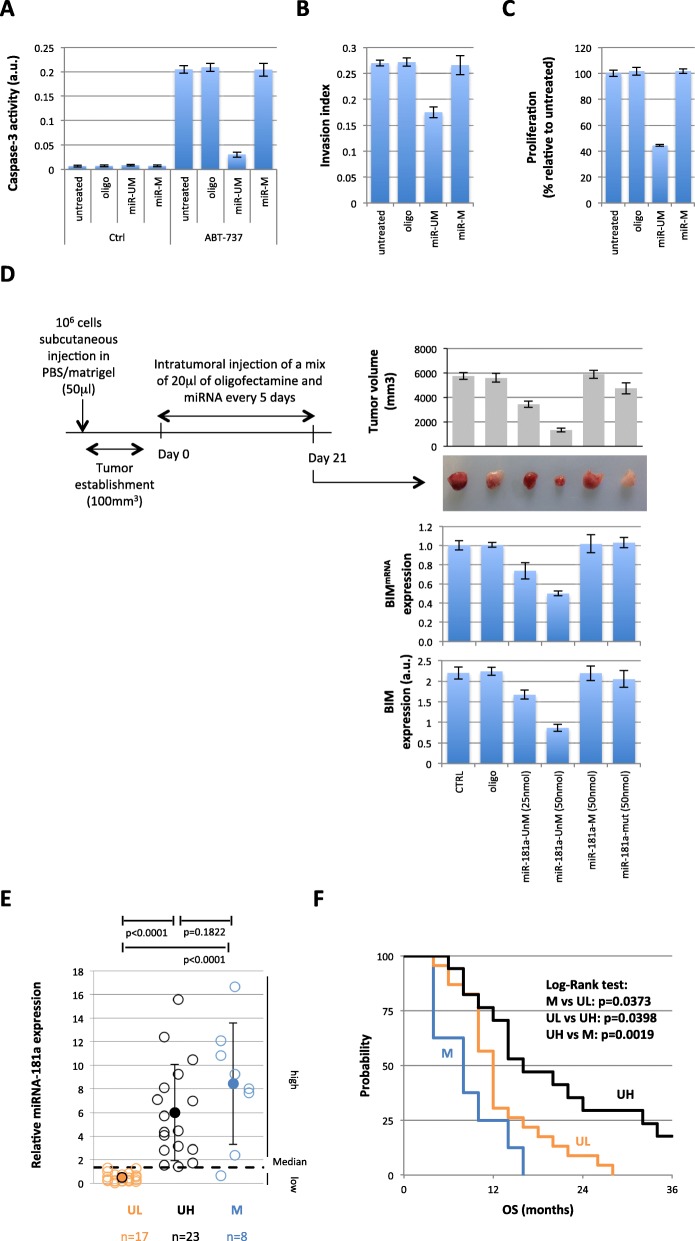
The presence of 5mC in miRNA-181a-5p abolishes its funtions. **a** Caspase-3 activity was measured to estimate apoptosis induction. Cells were co-treated with the indicated miRNA and ABT737 (1 μM) or control. Caspase-3 activity was determined using the Caspase 3 Assay Kit (Abcam, France). **b** Cell invasion determined by Collagen-Based Cell Invasion Assay (Millipore, France). **c** Cell Proliferation estimated by cell counting (Countess™ Automated Cell Counter (ThermoFisher, France)). **d** Impact of the methylation of miRNA-181a-5p on its tumor suppressor function and on BIM expression. The diagram illustrates the experimental procedures. Graphs illustrate the results obtained from 5 mice in each experimental condition. Pictures are representative of tumors obtained for each treatment. BIM expression was quantified using ELISA. **e** Graph illustrates the stratification of GBM patient samples according to their miRNA-181a-5p expression and methylation levels. Blue, open circles correspond to the patients whose miRNA-181a-5p was unmethylated and highly-expressed (UH). Red, open circles represent the patients with low expression of an unmethylated miRNA-181a-5p (UL). Red, closed circles represent the patients with a methylated miRNA-181a-5p (M). **f** Overall survival rates in the GBM patient subgroups (Kaplan-Meier) according to miRNA-181a-5p expression and the methylation status as described in Fig. 3i. Patients presenting low expression of an unmethylated miRNA-181a-5p (UL) and a methylated miRNA (M) were included in the same subgroups as these two “signatures” had a low effect on BIM

BIM is involved in implementing apoptosis by interacting with antiapoptotic Bcl-2 or Bcl-xl. We analyzed the impact of the cytosine methylation of miRNA-181a-5p on the apoptotic response to an inhibitor of Bcl-2, ABT-737 [25]. The apoptotic response, assessed by the caspase-3 activity, was abolished by cytosine methylation of miRNA-181a-5p (Fig. 5a). miRNA-181a-5p has been shown to decrease the proliferation and invasion of GBM cells (U87), and addition of cytosine methylated miRNA-181a-5p also affected this function (Fig. 5 b and c).

### Cytosine-methylated miRNA-181a-5p loses its tumor suppressor function and is associated with a poor prognosis factor in glioma

miRNAs may function as oncogenes or tumor suppressors. miRNA-181a-5p has been described as oncogenic in some types of cancer and as a tumor suppressor in others. In GBM, although 20–30% of GBMs underexpressed miRNA-181a-5p compared to normal brain, the oncogenic or tumor suppressor role of miRNA-181a-5p has not been clearly established [26] To clarify this point, we investigated whether or not the unmethylated form of miRNA-181a-5p could act as a tumor suppressor in GBM. For this purpose, human A172 gliomas were transplanted into nude mice and miRNA-181a-5p was directly injected into the subcutaneous tumors as described in Fig. 5d. Mice (*n* = 5 per group) were challenged-treated either with a low dose of lipofectamine-mediated unmethylated miRNA-181a-5p or a higher dose of unmethylated miRNA-181a-5p. Two controls were used with an in situ injection of PBS in the mock treated group and that of lipofectamine-mediated scrambled oligonucleotide in a second control group. A significant dose dependent-decrease in tumor volume was observed in the groups treated with miRNA-181a-5p, demonstrating the tumor suppressor role of miRNA-181a-5p (Fig. 5d). Next, we studied the impact of methylation of miRNA-181a-5p on its tumor suppressor function. For this purpose, five mice were treated with a high dose of methylated miRNA-181a-5p compared to its corresponding unmethylated form. Figure 5d shows that 5mC in miRNA-181a-5p abrogated its tumor suppressor function. Similarly, the cytosine mutation abrogated the tumor suppressor function of this miRNA in the control group. Consequently, our results confirmed the role played by cytosine-10 and -16 of miRNA-181a-5p in its function.

In addition, BIM expression was reduced in tumors treated with unmethylated miRNA-181a-5p, while BIM expression was unchanged in tumors treated with methylated or mutated miRNA-181a-5p. We thus conclude that methylated miRNA-181a-5p lost its repressor function against BIM (Fig. 5d).

We then evaluated whether or not the presence of methylated miRNA-181a-5p, as well as the low expression of miRNA-181a-5p, was associated with poor prognosis in glioma, while conversely, high expression of unmethylated miRNA-181a-5p may be associated with a good survival rate. Expression and methylation levels were thus analyzed in a collection of 48 GBM patients divided into two groups, based on the their expression and methylation levels of miRNA-181a-5p (**Supplementary Table T2**). Tumors from 17 patients expressed low levels of unmethylated miRNA-181a-5p (equal to or lower than the median value of miRNA-181a-5p expression, UL), tumors from 8 patients have a methylated miRNA-181a-5p (M), and tumors from 23 patients expressed a high level of unmethylated miRNA-181a-5p (higher than the median value of miRNA-181a-5p expression, UH) (Fig. 5e). The survival curves were estimated using the Kaplan-Meier method and compared with the Log-Rank test. Significant differences were observed between all patient subgroups (Fig. 5f). The low level of expression and the cytosine-methylation level of miRNA-181a-5p were associated with a poor survival prognosis (median: 12.4 and 8.5 months, respectively), and the high expression of unmethylated miRNA-181a-5p was associated with a more favorable survival prognosis in GBM (median: 16.5 months).

## Discussion

**Fig. 6:**
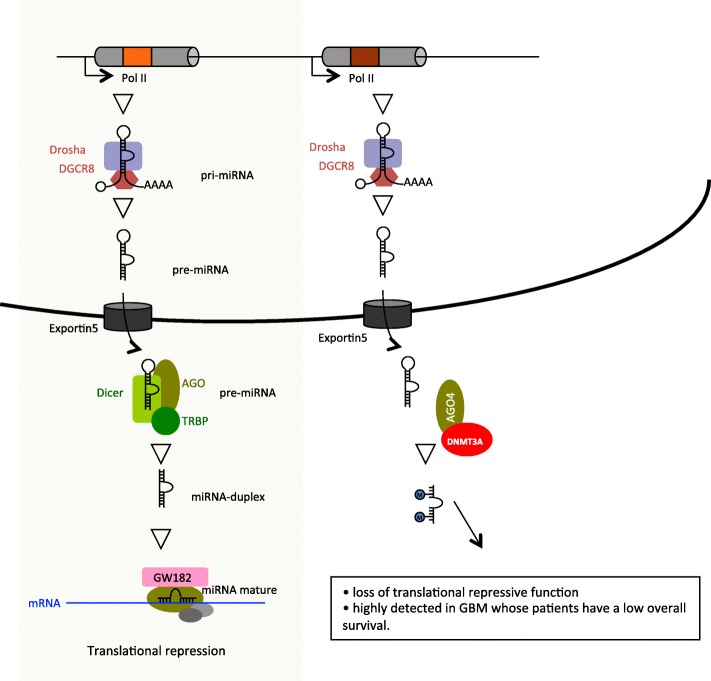
Representation of the miRNA methylation pathway compared to the canonical pathway of miRNA biogenesis (grey box). The miRNA biogenesis canonical pathway includes the production of the primary miRNA transcript (pri-miRNA) by the RNA polymerase II, and cleavage of the pri-miRNA by the microprocessor complex Drosha–DGCR8 in the nucleus. The resulting precursor hairpin, the pre-miRNA, is exported from the nucleus by Exportin-5. In the cytosol, the RNase Dicer in complex with the double-stranded RNA-binding protein TRBP cleaves the pre-miRNA hairpin to its mature length. The functional strand of the mature miRNA is loaded together with Argonaute and GW182 proteins into the RNA-induced silencing complex (RISC), where it guides RISC to silence target mRNAs and promote translational repression. The miRNA methylation pathway involves the AGO4/DNMT3A-including complex that catalyzes miRNA methylation in the cytosol, leading to the inhibition of its repressive function

Base modifications in miRNA is an emerging research area in the epitranscriptomics field. Our study shows the presence of cytosine methylation in mature miRNA. Thus, the present study provides experimental evidence and clinical data supporting the hypotheses that: i) miRNA can be methylated at cytosine residues by DNMT3A/AGO4-including complexes; ii) the presence of 5-methylcytosine (5mC) in miRNA abolishes their repressive function towards the gene expression; and iii) miRNA methylation is associated with poor prognosis in glioma (Fig. 6). The presence of 5mC in miRNA was supported by five different approaches: HPLC-UV, measure of DNMT3A-mediated incorporation of radio-labeled methyl group in miRNA, detection of 5mC in miRNA by ELISA and dot blot, miRNA immunoprecipitation by 5mC antibody (miRIP-5mC/Array), and miRNA bisulfite NGS. Of these experiments, two (HPLC-UV and Bisulfite sequencing) are currently considered to be the “gold standard” methods for quantifying and/or detecting 5mC in DNA and RNA [27–29]. Besides Xu et al. (2017) have recently used the HLPC-UV method to analyze base modifications to RNA [30]. To avoid any methodological bias, the antibody-based approaches (ELISA, dot blot and miRIP-5mC/Array) were carried out using different antibodies produced by four different companies (anti-5mC#Epigentek, anti-5mC#Active Motif, anti-5mC#Abcam and anti-5mC-Diagenode) and described in the literature. In addition to the manufacturer’s validations, “in house” validation of each antibody was performed and no signal was detected in ELISA, dot blot or 5mC-miRIP in the presence of unmethylated mimetic miRNA (**Fig. S13**). By performing miR-BSeq and miRIP-5mC/Array analyses, our work permits to identify miRNA-16-5p, miRNA-181a-5p, miRNA-181b-5p, miRNA-181d-5p and miRNA-210-3p as cytosine-methylated miRNAs in U87 cells. miRIP-5mC/Array analyses perfomred on other glioblastoma cell lines supported that the cytosine methylation of miRNA-181a-5p was commonly observed in GBM. This identification is based on three points: i) the primers specificity used in the miR Array (Qiagen, France), ii) the sequence matching against the mature miRNA sequences were downloaded from miRBase-21, and iii) the absence of sequence matching with piRNA and tRF (via the use of two databases: http://www.regulatoryrna.org/database/piRNA/ and http://genome.bioch.virginia.edu/trfdb/). As to the putative overlapping between miRNA and tRF, we noted that the five cytosine-methylated miRNA in our study is not included in the miRs overlapping with tRF [31]. Thus, our data is one of the first to demonstrate the presence of 5mC in miRNA in human cells using a panel of five different methods. Cytosine-methylation of mature miRNA appears to be conserved across species as in Arabidopsis its role is to protect miRNAs from a 3′-end uridylation activity [32].

By identifying that pre-miRNA and pri-miRNA can be phospho-dimethylated and 6-adenosine-methylated, Xemalce et al. (2012) and Alarcón et al. (2015) were the first to publish that an expressed form miRNA can possess modifications [8, 9] (Supplementary Table T3). Berulava et al. (2015), Pandolfini et al. (2019) and Konno et al. (2019) identified that mature miRNA can be 6-adenosine methylated, 7-guanosine methylated and 5-cytosine-methylated [10, 13, 14]. Our data complete these findings by showing that 5-cytosine-methylation negatively regulates miRNA functions. Four distinct experiments/observations support this hypothesis: i) a cytosine methylated miRNA that did not repress the expression of one of its targeted proteins; ii) a cytosine methylated miRNA that did not repress luciferase expression from a 3’UTR reporter plasmid; iii) the correlation between the presence of cytosine methylated miRNA and the high expression of one of its targeted proteins; iv) the fact that a miRNA with a tumor suppressor function lost its function after cytosine methylation.

To date, three interplays between cytosine methylation and the miRNA regulation have already been described: miRNA gene expression is regulated by the presence of methylated cytosine in its promoter and/or coding region [33], miRNA can affect DNA methylation by targeting the expression of a DNMT [34, 35], and miRNA can inhibit DNA methylation [36]. Thus, by describing that 5mC in miRNA induces the loss of their repressive function against gene expression our study provides a new interplay between cytosine methylation and miRNA regulation. From a more mechanistic point of view, our findings indicate that the cytosine methylation of miRNAs inhibits miRNA/mRNA duplex formation. In our study, this point is supported by 2 distinct experiments analyzing the formation of miRNA/mRNA interactions: CLIP-qPCR and Biotin-tagged miRNA or 3’UTR/mRNA.

The identification of DNMT3A/AGO4 as a miRNA “cytosine-methylator” is supported by four approaches affecting the integrity of the DNMT3A/AGO4 interaction (siRNA directed against DNMT3A and AGO4, disrupting antibody directed against AGO4, and correlation studies between cytosine-methylation of miRNA and DNMT3A/AGO4 levels), and the DNMT3A/AGO4 interaction integrity is itself analyzed by three distinct experiments (co-immunoprecipitation, pull-down assay and P-LISA). By identifying DNMT3A/AGO4 as a key regulator of miRNA methylation, our data also reinforce the interplay between miRNA and cytosine methylation. AGO4 has already been identified as a major actor in miRNA biogenesis, and DNMT3A as a crucial enzyme regulating cytosine methylation in DNA [37, 38]. The implication of DNMT3A in a process methylating “a single stranded oligonucleotide structure” could be surprising as this enzyme is well-known for methylating double-stranded DNA [39, 40]. However, Yokochi et al. (2002) and the present data support the idea that the monomeric form of DNMT3A has the ability to methylate a single-stranded oligonucleotide even if this methylation is 14 times less than that performed against a double-stranded oligonucleotide [41]. In addition, the presence of AGO4 increases (9 times) the DNMT3A-mediated methylation of miRNA. DNMT has previously been suspected of being involved in RNA methylation. Goll et al. (2006) and Jurkowski et al. (2008) demonstrated that DNMT2 methylated a “specific form of the RNA molecule” (tRNA) using its DNA methyltransferase-like catalytic mechanism [42, 43]. The implication of DNMT3A, a protein mainly known for its nuclear activity in DNA methylation, may appear surprising. However, several articles report that DNMT3A can be weakly cytoplasmic [44–48]. The “Atlas protein website (https://www.proteinatlas.org/ENSG00000119772-DNMT3A/tissue)” also reports that “DNMT3A localizes to the cytoplasm and nucleus “. Based on all these arguments, we propose that DNMT3A methylates miRNA that are recruited by AGO4.

It may be questioned if this process is specifically restricted to miRNA-181a-5p or could be considered as a general mechanism. We therefore studied several miRNA using Western blot and/or reporter 3′-UTR experiments that showed the cytosine methylation of miRNA-193a-5p and miRNA-451a abolished the repression of TP73 and Bcl-2 expression, respectively (Figures S14 and Figure S15). Consequently, DNMT3A/AGO4 is also involved in the cytosine methylation of miRNA-451a and miRNA-193a-5p as these parameters are correlated in a cohort of 32 GBM patients and the siRNA-mediated invalidation of DNMT3A or AGO4 decreased the percentage of methylated miRNA-451a and miRNA-193a-5p. As described for miRNA-181a-5p, cytosine methylation of miRNA-451a and miRNA-193a-5p abolished the modulation of cellular phenotypes regulated by their unmethylated form (Figures S14 and Figure S15). The cytosine methylation of miRNA-451a may also affect its tumor suppressor function (miRNA-193a-5p had no tumor suppressor effect in our study) (Figures S14 and Figure S15). Through the 3 examples considered, it appears that the cytosine methylation of miRNA acts as a negative regulator of miRNA functionality in a general manner. The low miRNA-181a-5p expression and methylation level of miRNA-181a-5p were associated with a poor prognosis factor in GBM patients. A similar observation was also made with miRNA-451a but not with miRNA-193a-5p (Figure S16). These observations are consistent with the fact that miRNA-181a-5p and miRNA-451a promote tumor suppression when they are administered in GBM, unlike miRNA-193a-5p.

Finally, BS sequencing data indicate that the cytosine methylation level of miR varies from 9 to 24%. Recently, Konno et al. (2019) reported a percentage of 5mC of miRNA-200-3p, miR-NA21-5p between 2.5 to 5.5% in a context of colorectal cancer and paired normal tissues [14]. It is worthwhile to mention that research reports identifying the presence of m6A and m7G in mature miRNA usually present their data in fold enrichment [10, 13]. Thus, our findings appear to be in line with the report from Konno et al. (2019) [14]. One might wonder on the reason of this low methylation percentage. In other term, this first finding opens multiple other questions on the stability of cytosine-methylation of miRNA (Can cytosine-methylation been erased?), on its role on the putative presence of other base modifications, on its influence of the miRNA « capture » by circRNA, or on its impact on the miRNA exporting in extracellular vesicles such as exosomes. All these questions highlight the need of deeper investigations of miRNA methylation biology.

## Conclusion

This first demonstration of the miRNA cytosine-methylation process (and not on a miRNA promoter/gene) in human cells opens a new era in the understanding of pathophysiological processes involving miRNA. A better understanding of its functional impact in malignant transformation will improve the development of successful therapeutic strategies as well as provide therapeutic targets for restoring normal miRNA function in cancer cells.

## Supplementary information

**Additional file 1.** Schematic representation of BS experiments.

**Additional file 2.** Supplementary figures and tables.

**Additional file 3.** Statistical analysis of BS experiments.

**Additional file 4.** Results of BS experiments.

**Additional file 5.** Sequence of selected miRNA-BS.

**Additional file 6.** Results of selected miRNA-BS.

**Additional file 7.** Results of 5mC-RIP-Array analyses.

**Additional file 8.** Extended experimental procedures.

## Data Availability

Raw sequencing data from this study has been deposited into ArrayExpress.

## Abbreviations

5mC: 5-methylcytosine
AGO4: Argonaute-4
CLIP-qPCR: Cross-linking immunoprecipitation and qPCR
DNMT3A: DNA methyltransferase 3A
GBM: Glioblastoma multiforme
miRNA: microARN
tRNA: Transfert RNA
