# Supplementary figures and images for: Cytosine methylation of mature microRNAs inhibits their functions and is associated with poor prognosis in glioblastoma multiforme
Source: Mol Cancer. 2020 Feb 25;19:36. doi: 10.1186/s12943-020-01155-z (PMC7041276; doi:10.1186/s12943-020-01155-z)

# NGS sequencing

# Mapping & Filtering

# Statistics

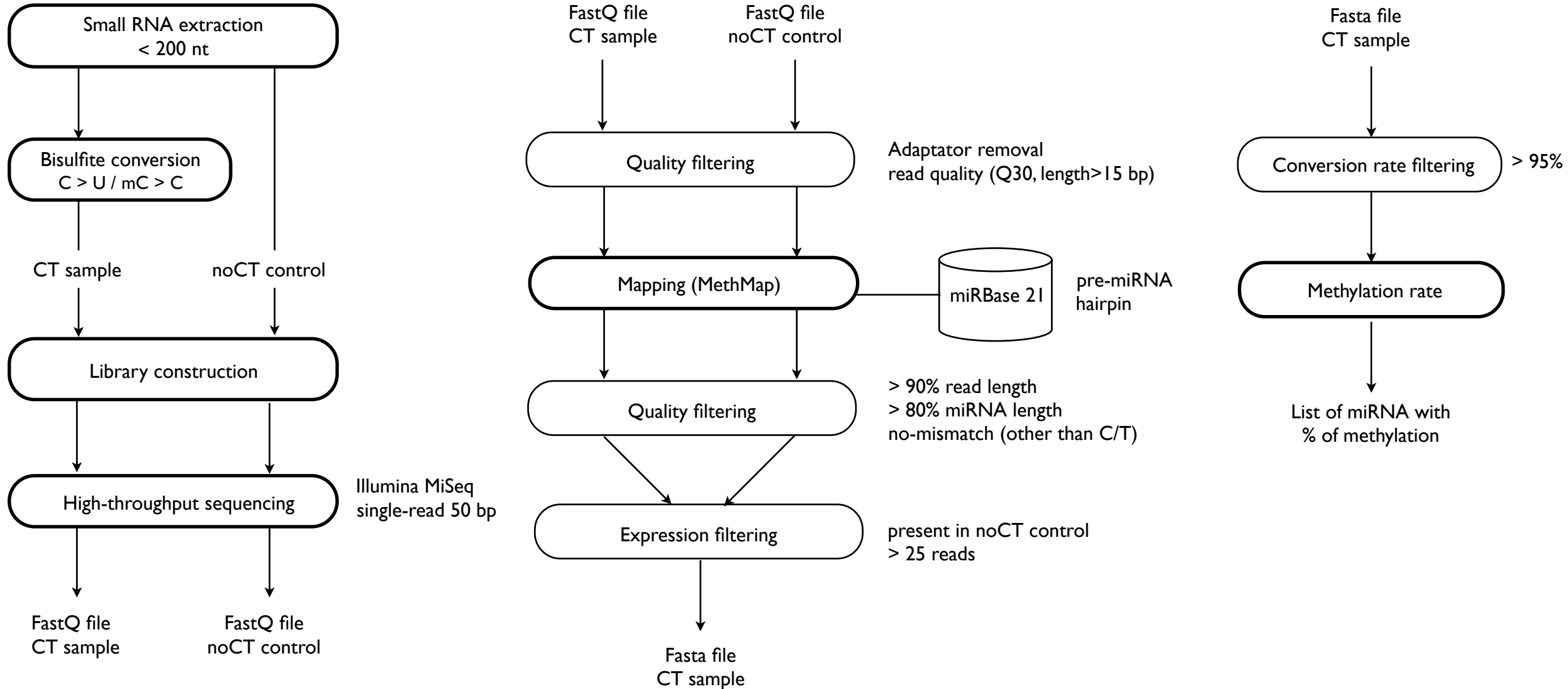

Supplement: Supplementary file 1 — Additional file 1. Schematic representation of BS experiments. [file 12943_2020_1155_MOESM1_ESM.pdf]
