# Supplementary material for: Cytosine methylation of mature microRNAs inhibits their functions and is associated with poor prognosis in glioblastoma multiforme
Source: Mol Cancer. 2020 Feb 25;19:36. doi: 10.1186/s12943-020-01155-z (PMC7041276; doi:10.1186/s12943-020-01155-z)
Supplement: Supplementary file 2 — Additional file 2. Supplementary figures and tables. [file 12943_2020_1155_MOESM2_ESM.pdf]

Supplementary Figures and tables

Characteristics		Patients (n=32)
Age (years)<br>Median (range)		63 (48;76)
Sex		
	Male (%)	18 (56)
	Female (%)	14 (44)
Karnofsky performance score		
	60-80 (%)	11 (34)
	90-100 (%)	21 (66)
Survival time (months)		
Median (range)		12 (5;50)
Extent of surgery (%)		
	Biopsie	0
	Partial resection	0
	Complete resection	32 (100)
MGMT methylation		
	Methylated (%)	15 (47)
	Unmethylated (%)	17 (53)
IDH1 mutation		
	Wild-type (%)	31 (97)
	Mutated (%)	1 (3)

Supplementary Table T1.

Characteristics		Patients (n=48)
Age (years)<br>Median (range)		62<br>(48;76)
Sex		
	Male (%)	26 (54)
	Female (%)	22 (46)
Karnofsky performance score		
	60-80 (%)	16 (33)
	90-100 (%)	32 (67)
Survival time (months)		
Median (range)		13 (4;53)
Extent of surgery (%)		
	Biopsie	0
	Partial resection	0
	Complete resection	48 (100)
MGMT methylation		
	Methylated (%)	23 (48)
	Unmethylated (%)	25 (52)
IDH1 mutation		
	Wild-type (%)	47 (98)
	Mutated (%)	1 (2)

Supplementary Table T2.

Base modification detection	Methods	References
Detection of cytosine methylation in RNA	Bisulfite sequencing	Legrand et al. (2017) Genome Res. PMID: 28684555
Detection of Adenosine methylation in pri-miR	m6A Immunoprecipitatio n and RNA seq	Alarcon et al. (2015) Nature. PMID: 25799998
Detection of phospho-dimethylated pre-miR-145	Incorporation of radiolabelled groups	Xelmace et al. (2012) Cell. PMID: 23063121
Detection of cytosine methylation in RNA	Bisulfite sequencing	Schaefer et al. (2009) Nucleic Acids Res. PMID: 19059995
Detection of cytosine methylation in tRNA	Mass spectrometry	Goll et al. (2006) Science. PMID: 16424344
Detection of guanosine methylation in miRNA	Borohydride Reduction sequencing [BoRed-seq]	Pandolfini et al. (2019) Molecular Cell. PMID: 31031083
Detection of cytosine and adenosine methylation in miRNA	MALDI-TOF-MS	Konno et al. (2019) Nature Communications. PMID: 31467274

Supplementary Table T3.

A

source: https://www.genomics.agilent.com/article.jsp?pageId=1300003)

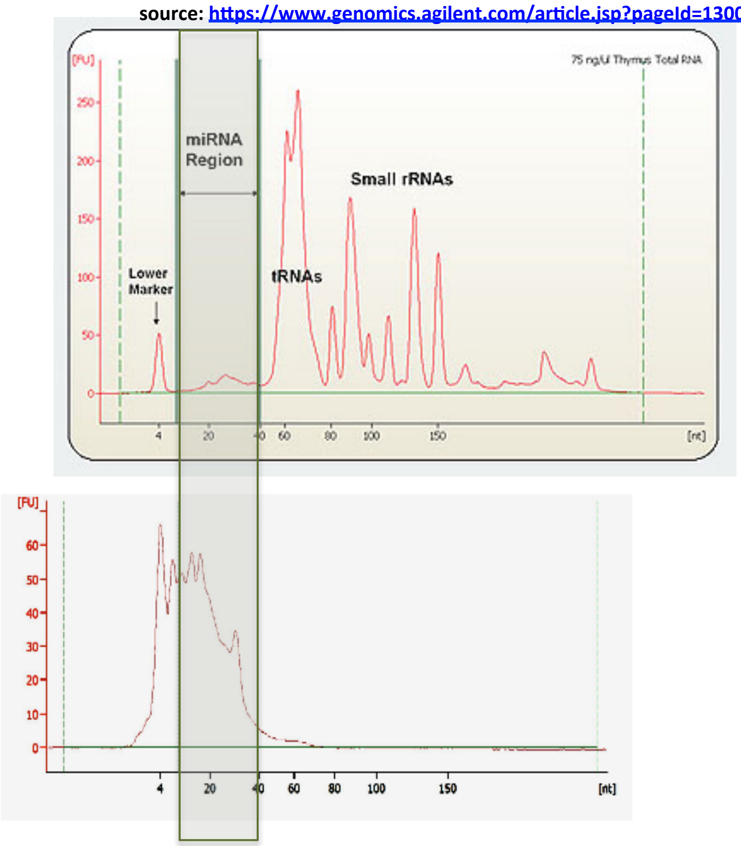

B

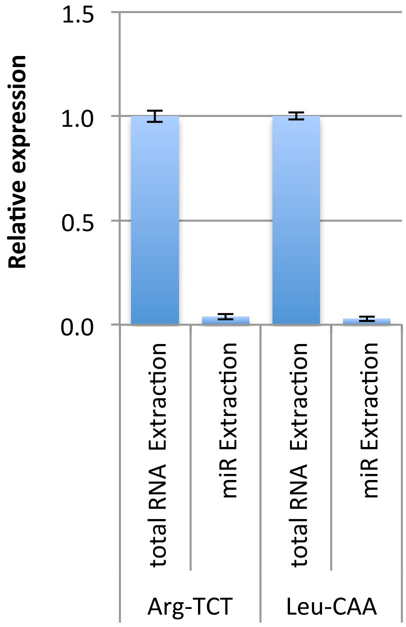

Figure S1. Experimentations illustrating the purity of our miRNA extractions.

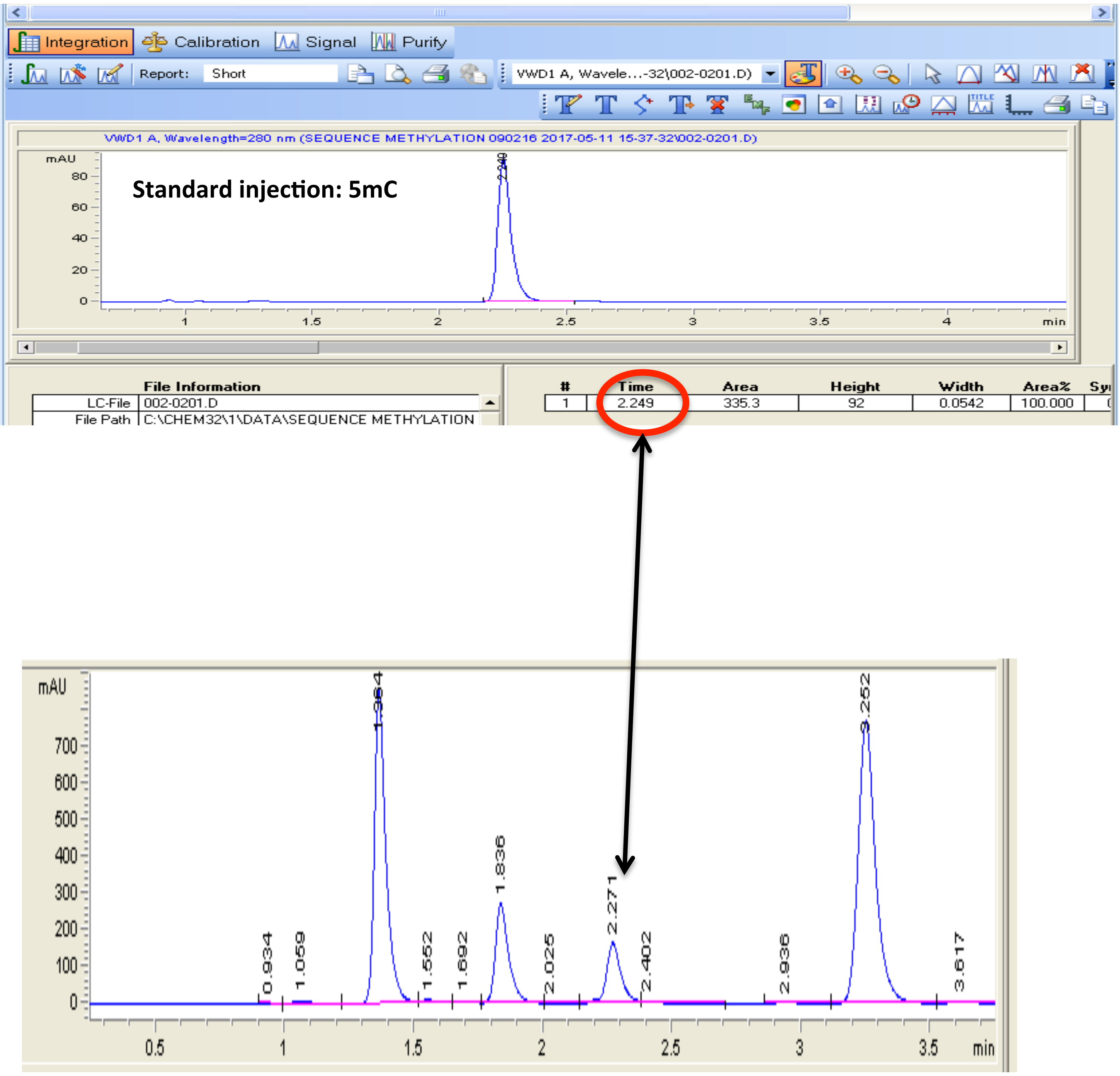

Figure S2. Calibration of HPLC for 5mC.

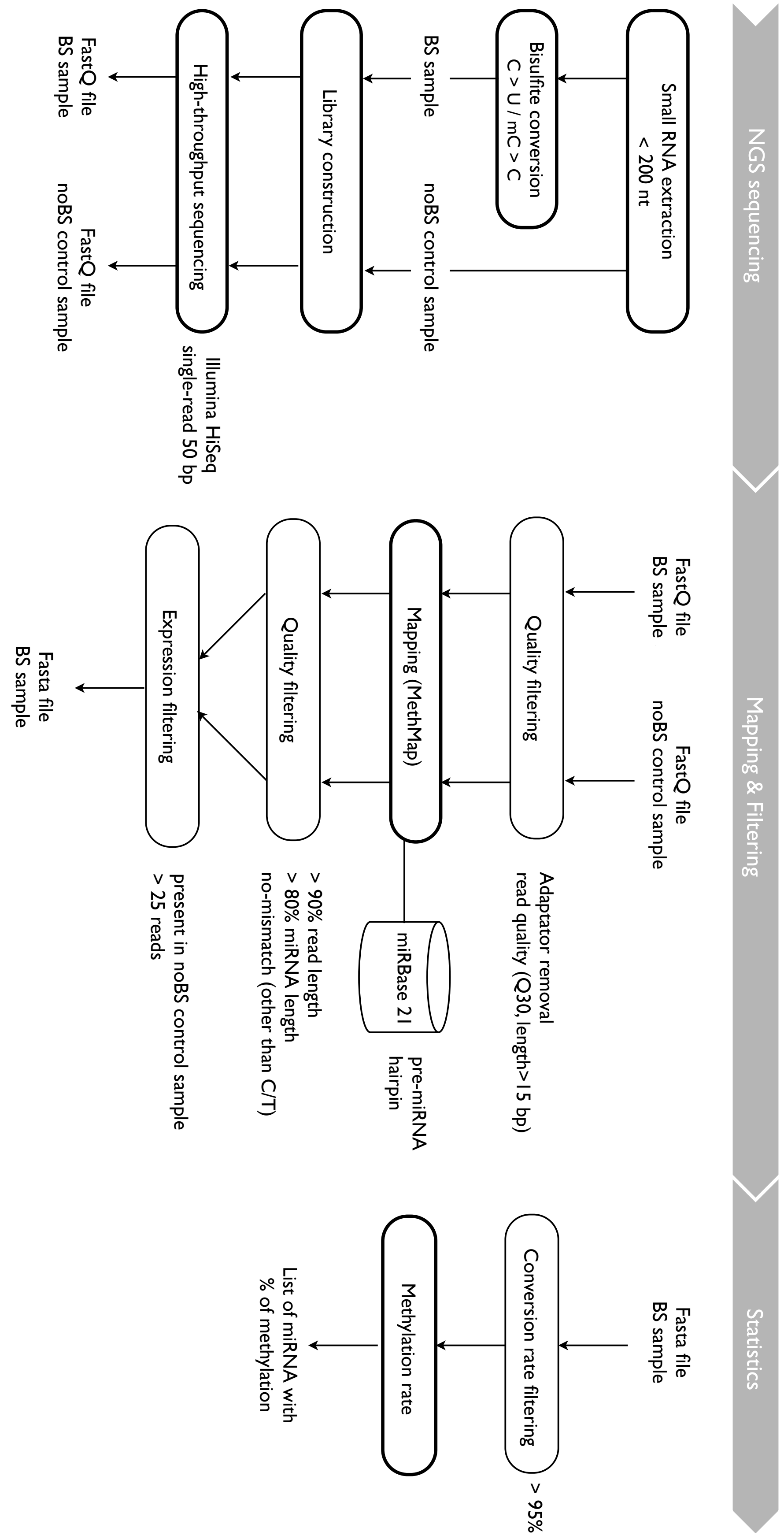

Figure S3. miRNA-Bseq workflow.

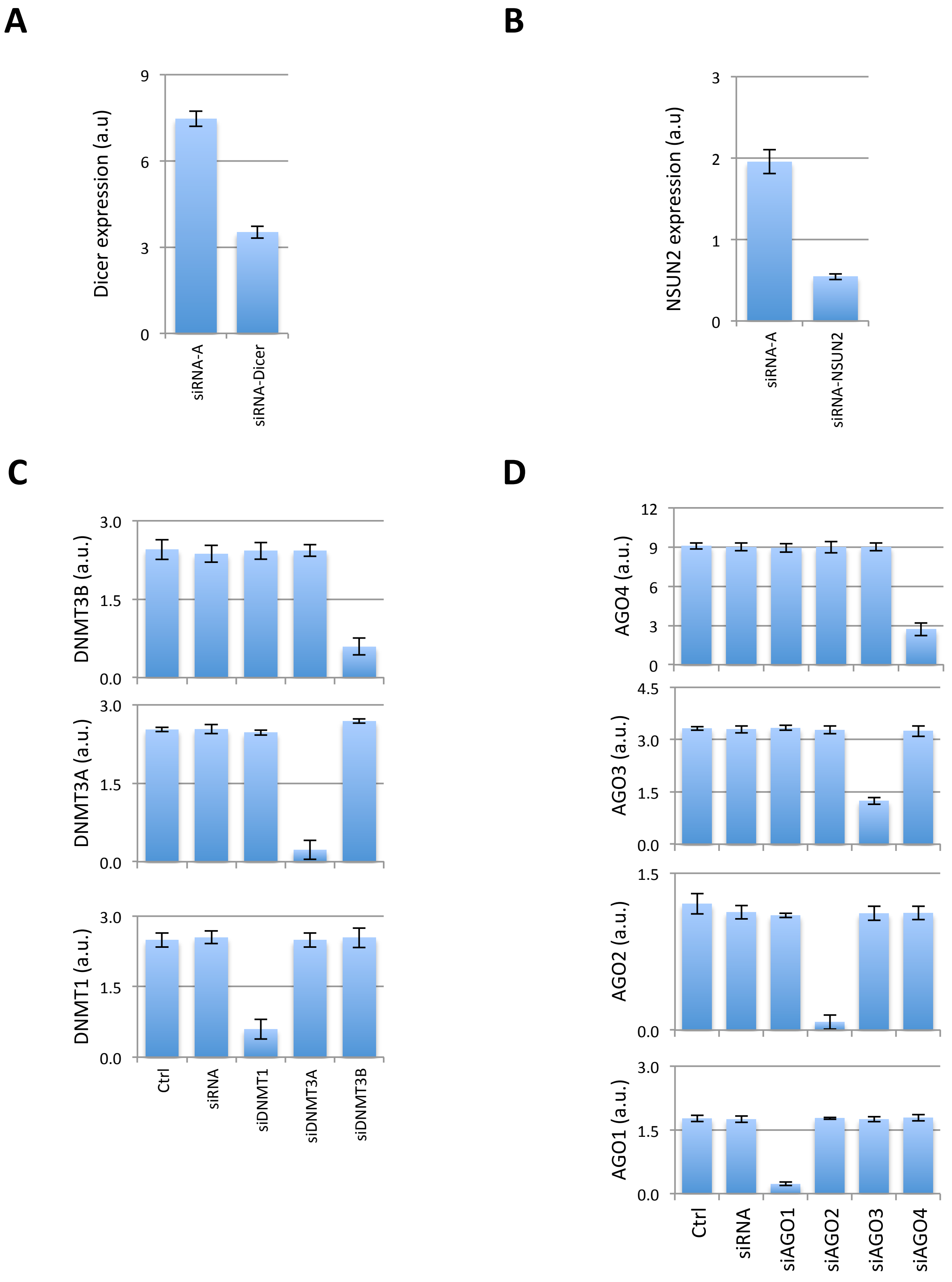

Figure S4. In-cell ELISA investigating the siRNA-mediated down-regulation alidation of Dicer, NSUN2, DNMT1/3A/3B and AGO1/2/3/4

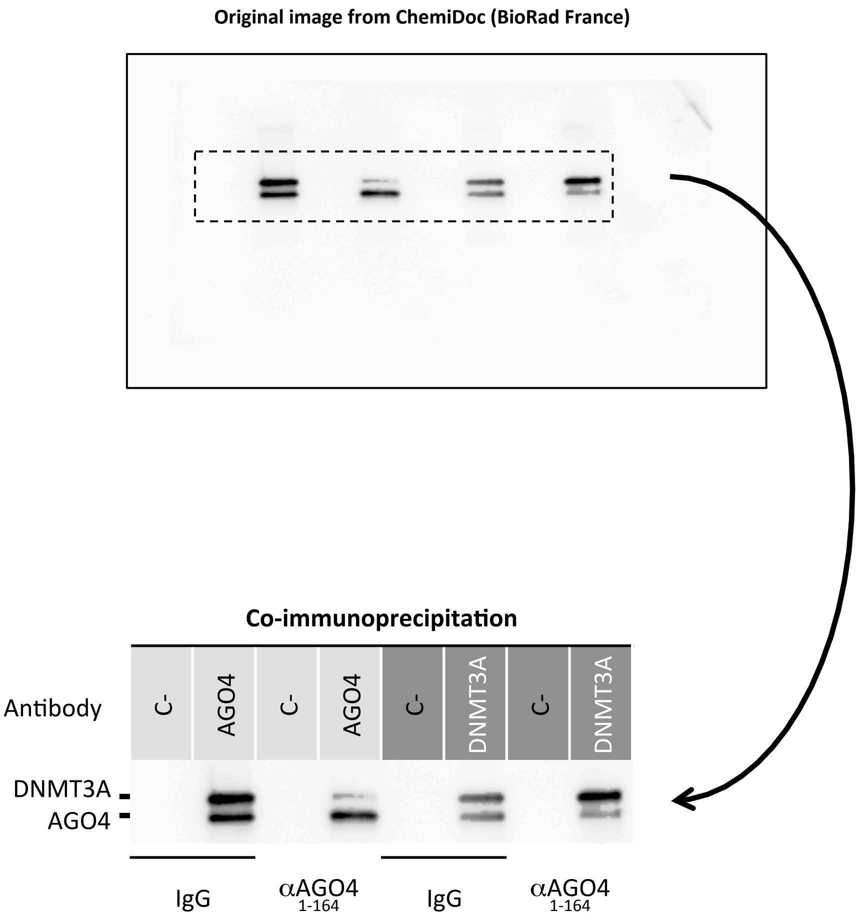

Figure S5. Original image of co-immunoprecipitation experiments.

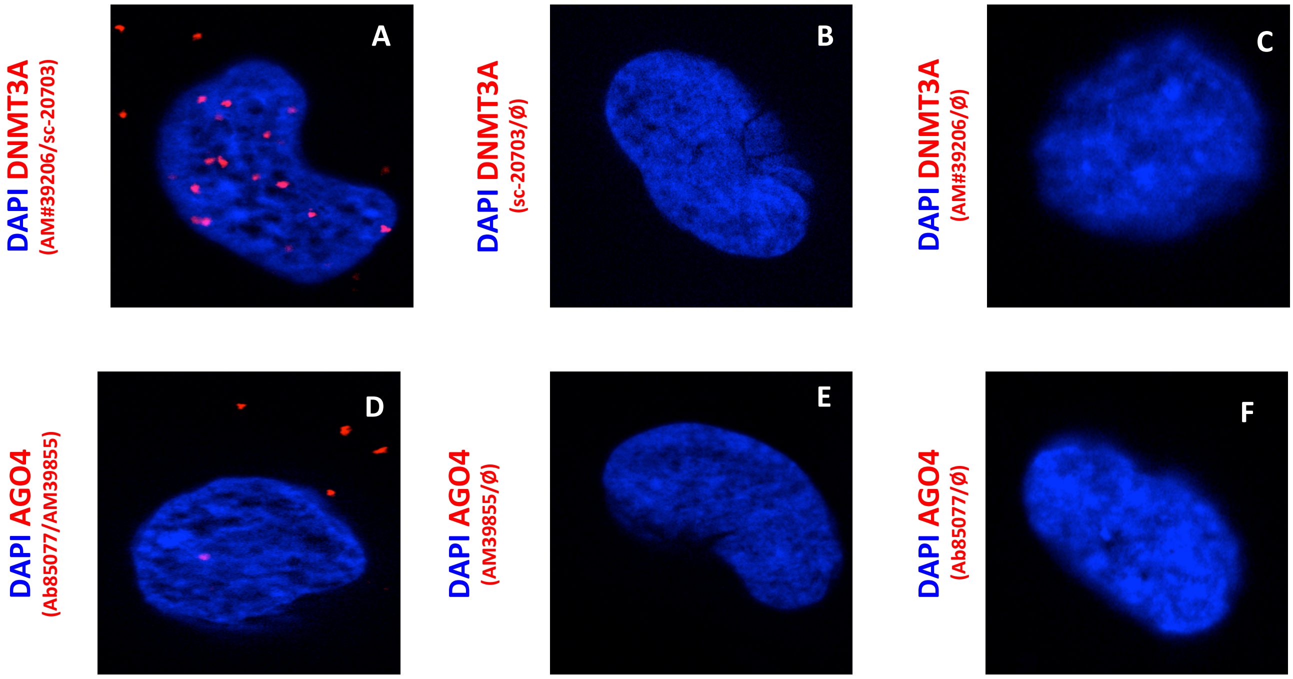

Figure S6. Validation of antibodies used in P-LISA experiments.

Original images from ChemiDoc (BioRad France)

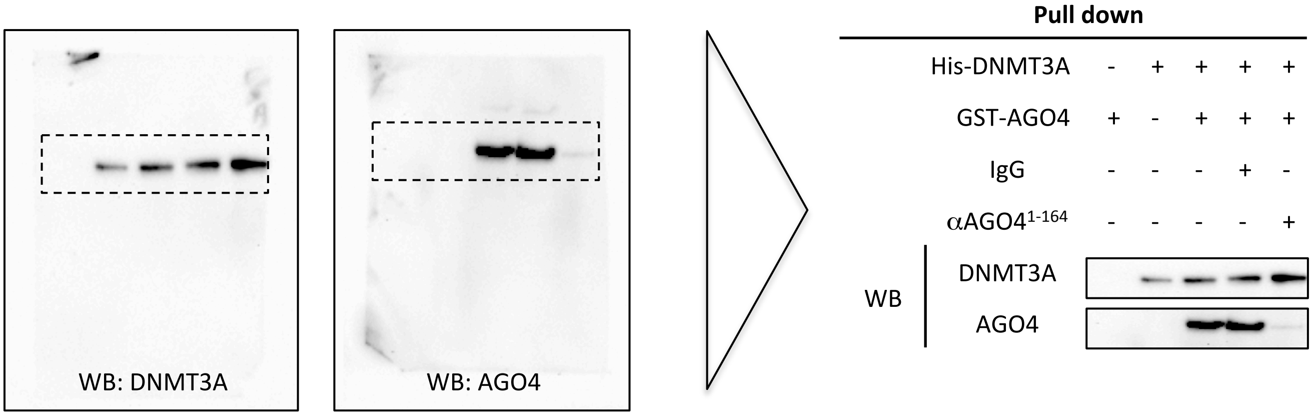

Figure S7. Original images of pull-down experiments.

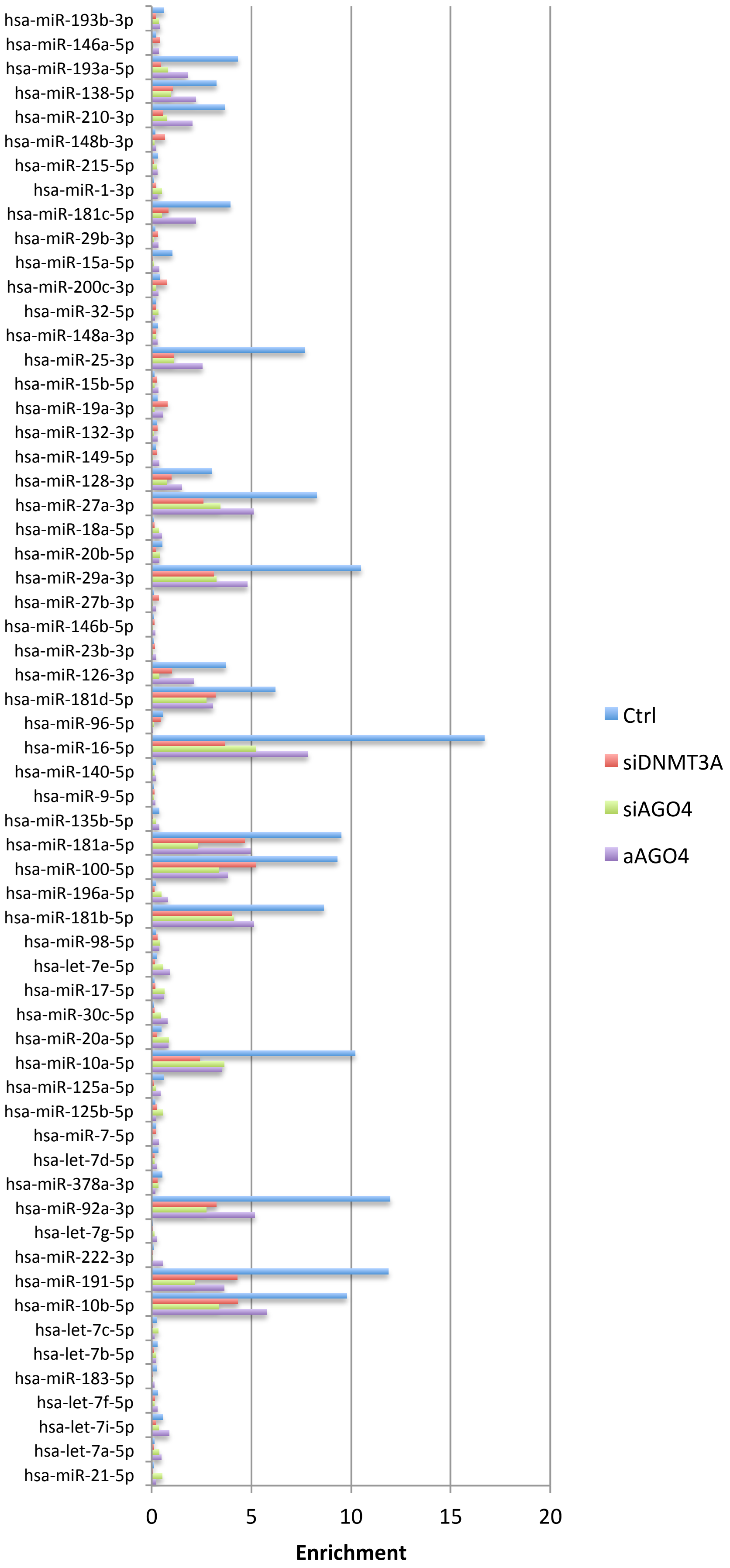

Figure S8. Impact of siDNMT3A or AGO4 and αAGO4$^{1\text{-}164}$ on the miR-Array profile

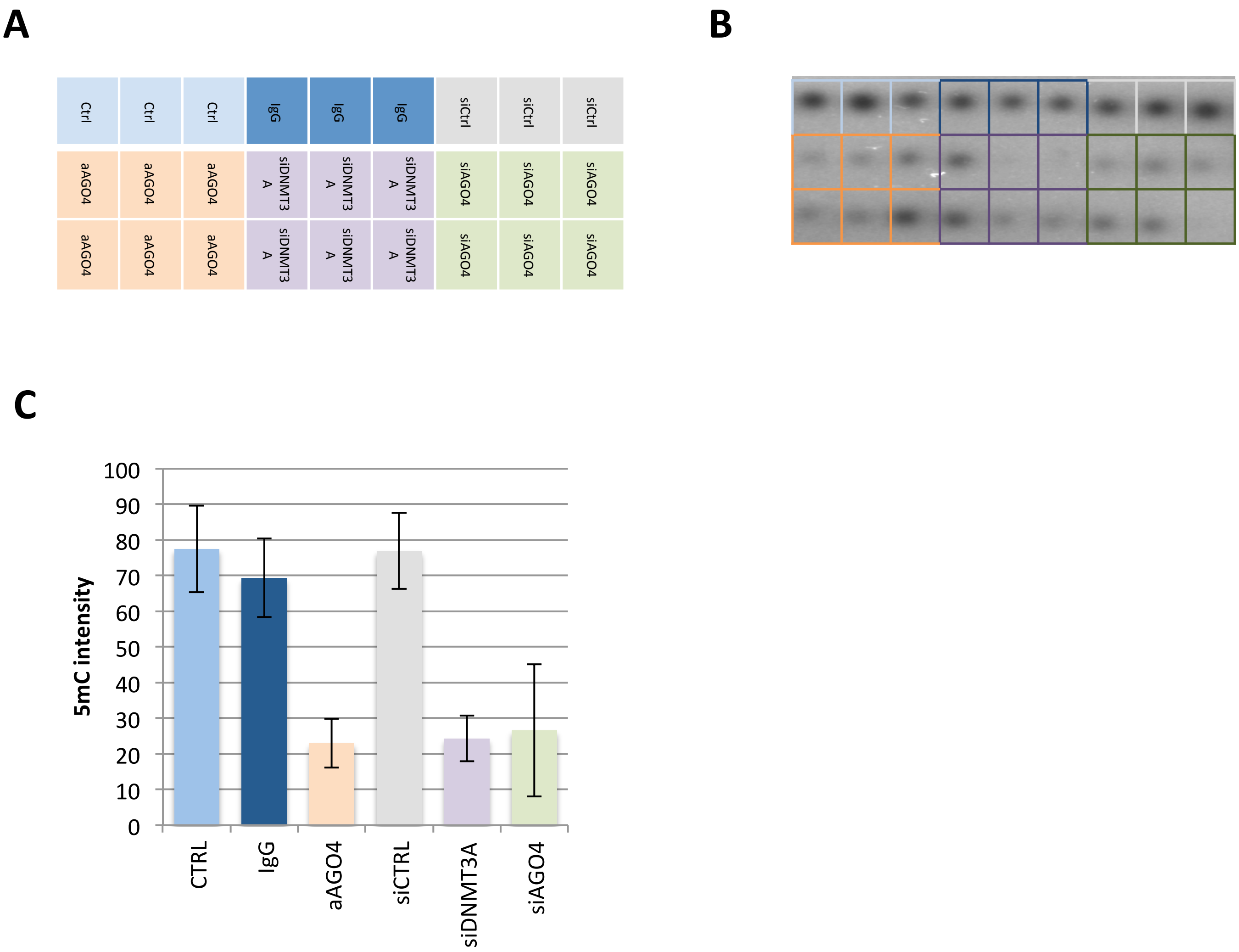

Figure S9. Dot blot illustrating the impact of the siRNA-mediated invalidation of DNMT3A and AGO4 and the use of the aAGO4$^{1\text{-}164}$ antibody on the global level of cytosine-methylation of miRNA.

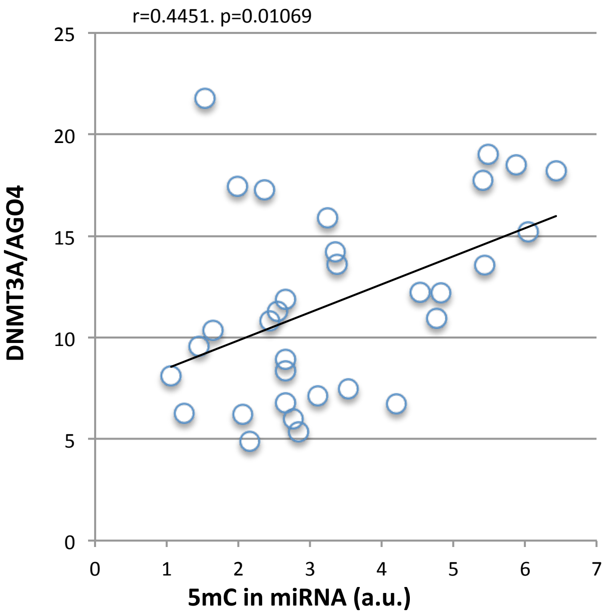

Figure S10. Correlation between the level of DNMT3A/AGO4 interactions and the global level of cytosine-methylation of miRNA

3’UTR BIM	A	U	G	U	A	A	G	U	G	U	U	G	U	U	U	G	A	A	G	G	U	A	A				
		I	I	I	I	I	I	I			I	I	:			I	I		:	I	:		I				
miRNA-181a (MIMAT0000256)	a	a	c	a	u	u	c	a	-	-	a	c	g	–	–	c	u	g	u	c	g	g	u	g	a	g	u
Position	1	2	3	4	5	6	7	8			9	10	11			12	13	14	15	16	17	18	19	20	21	22	23
Cytosine-methylated												X								X							
Mutated#1			u		c		a	g																			
Mutated#2												a								a							

Figure S11. Illustration of the he miR-181a binding site in the 3’UTR of Bim and the miRNA-181a mutant or methylated sequences.

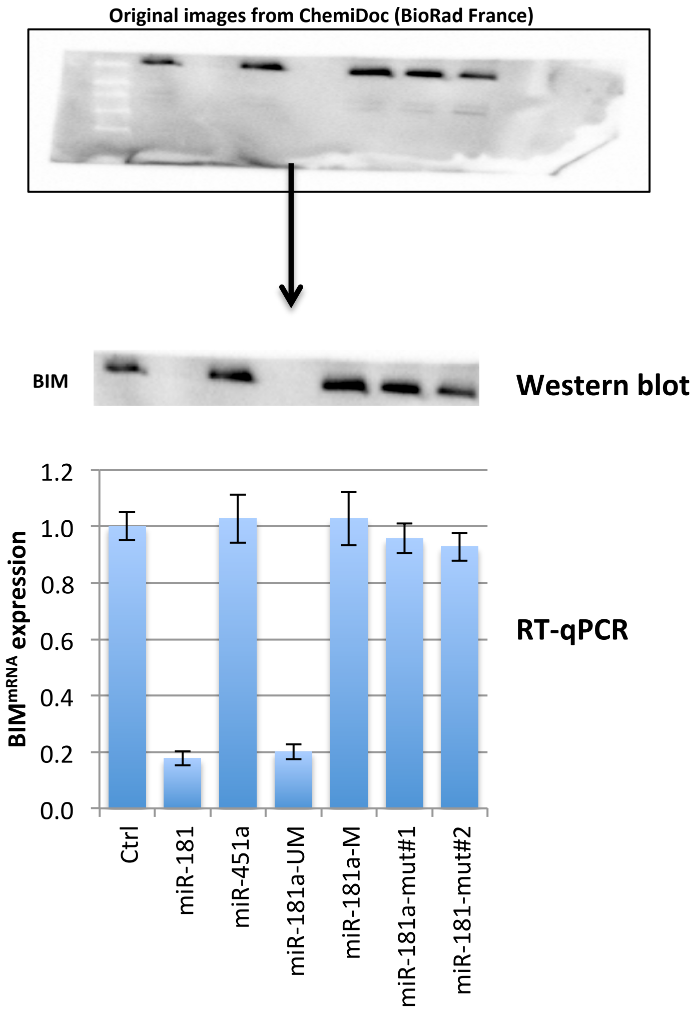

Figure S12. Western blot and RT-qPCR investigating the effect of miRNA-181a-5p on BIM expression at protein and mRNA levels.

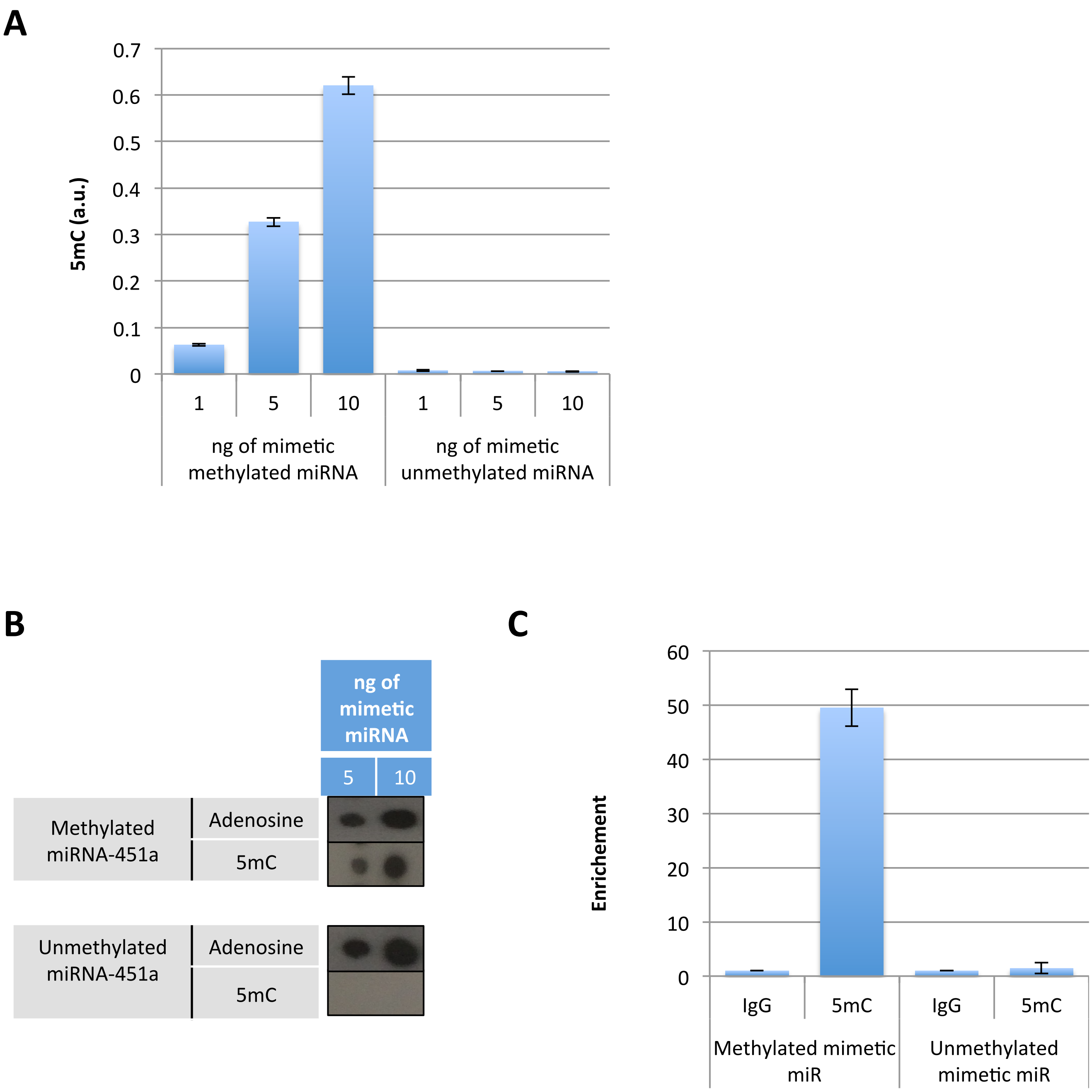

Figure S13. Validation of 5methylcytosine (5mC) antibodies used in our study.

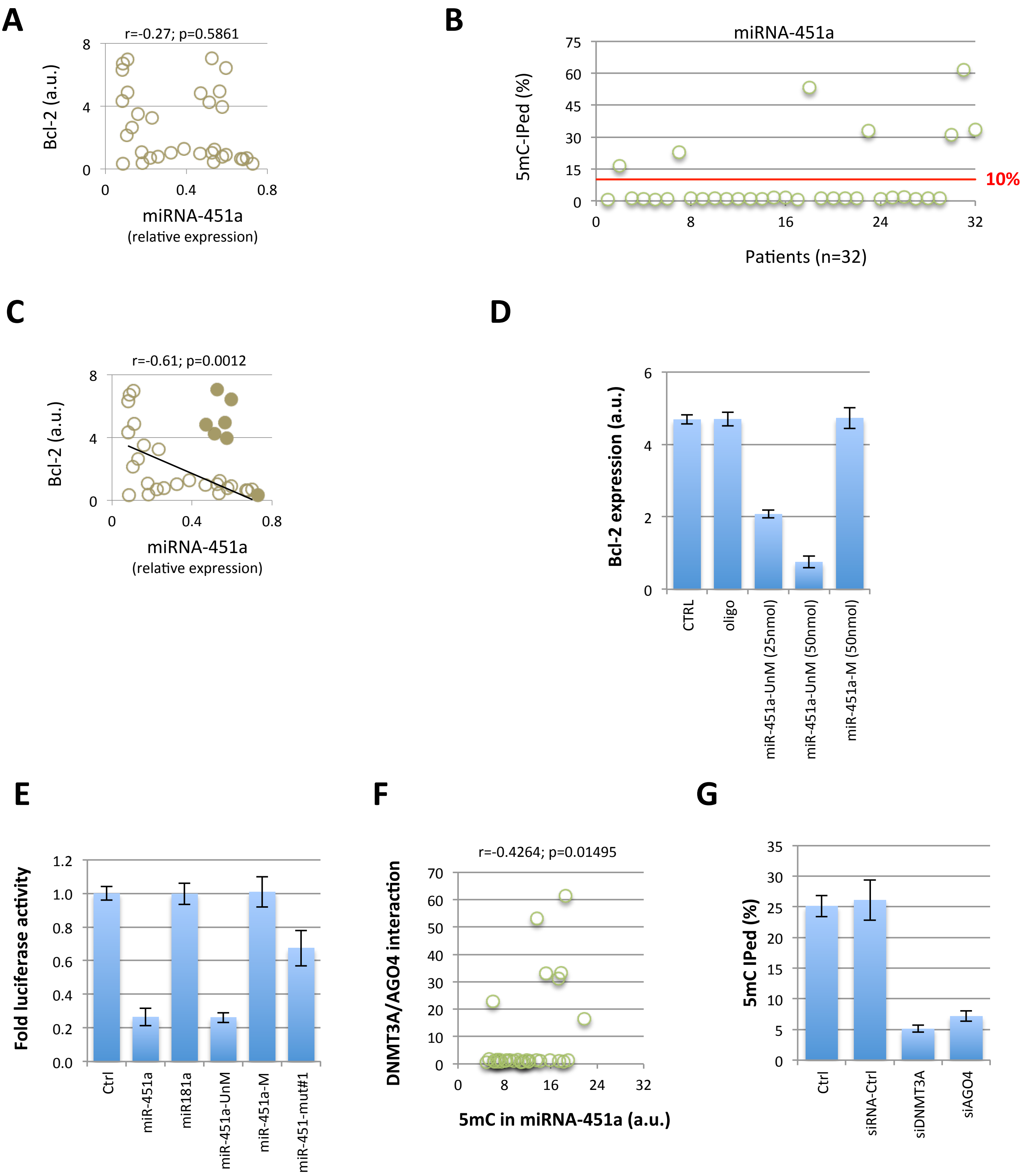

Figure S14: The presence of 5mC in miRNA-451a abolishes its functions

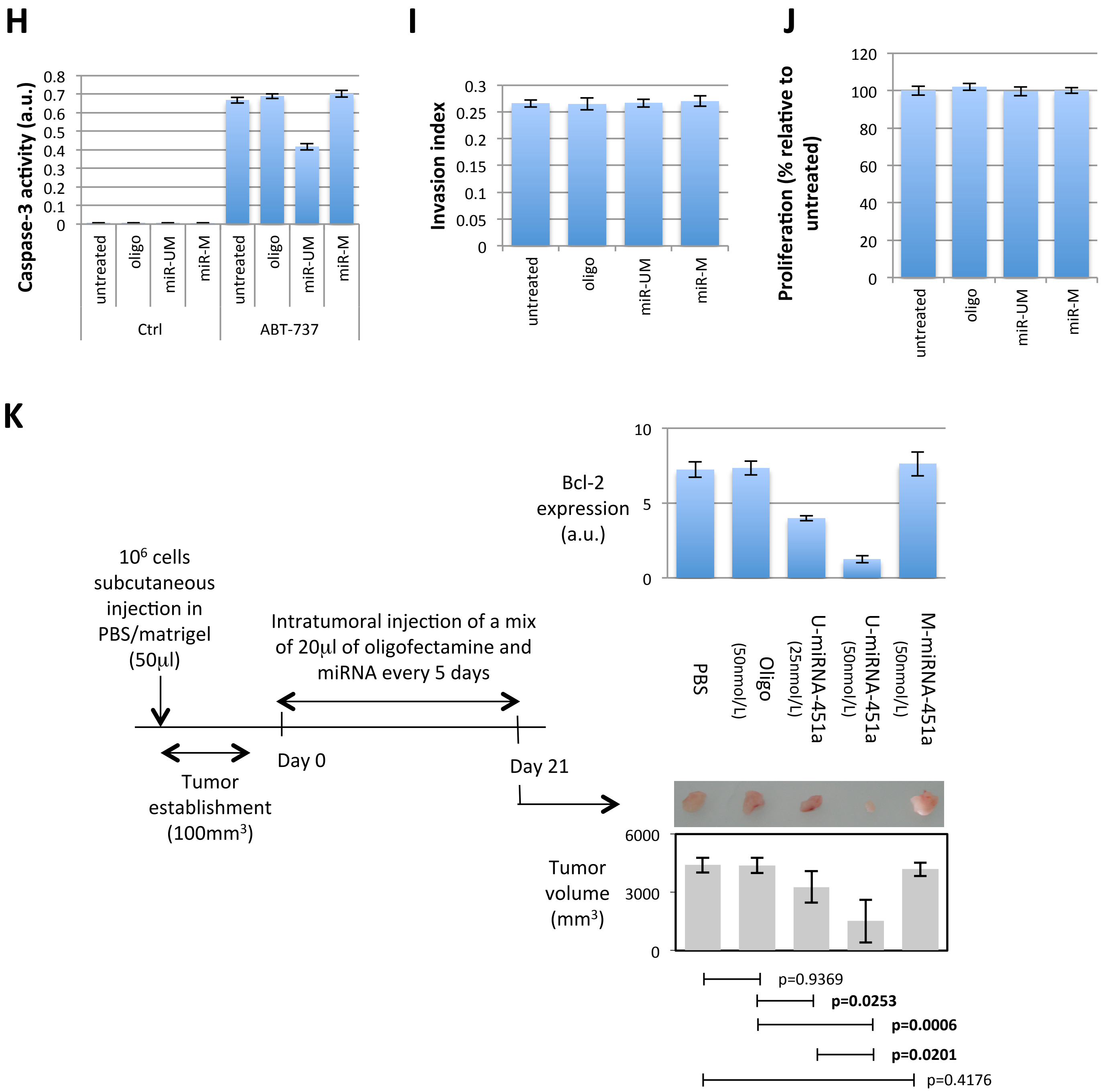

Figure S14: The presence of 5mC in miRNA-451a abolishes its functions

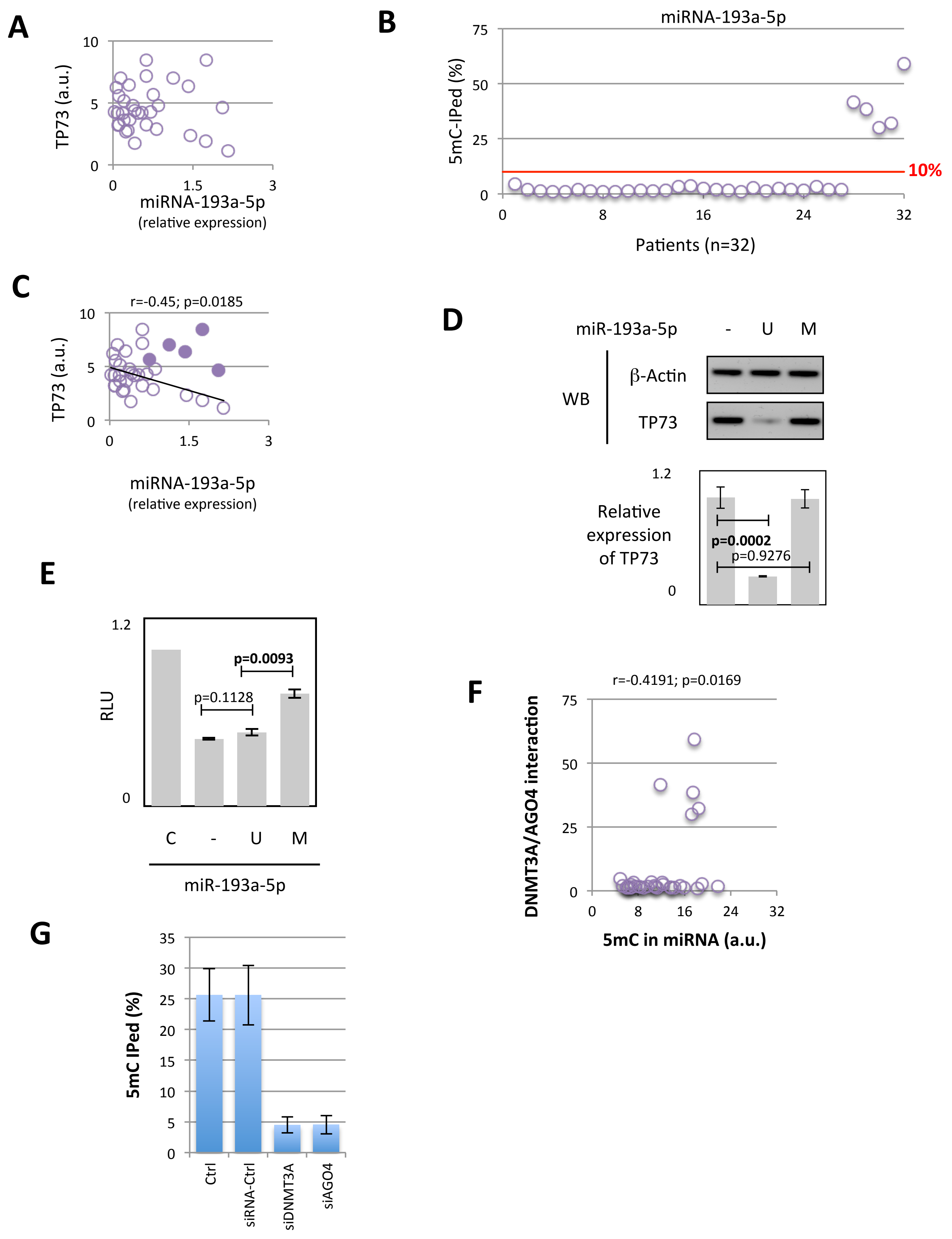

Figure S15: The presence of 5mC in miRNA-193a-5p abolishes its functions

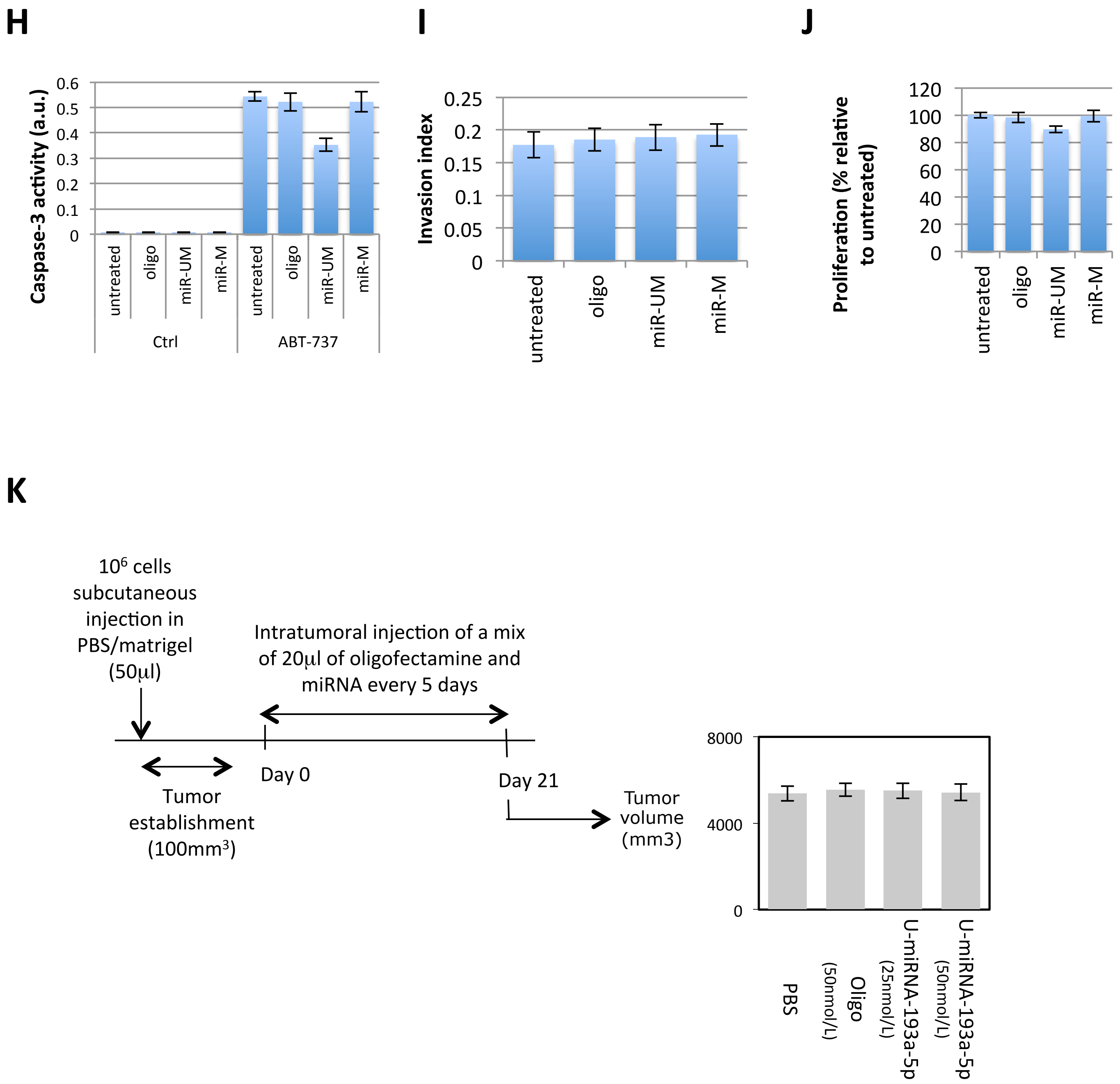

Figure S15: The presence of 5mC in miRNA-193a-5p abolishes its functions

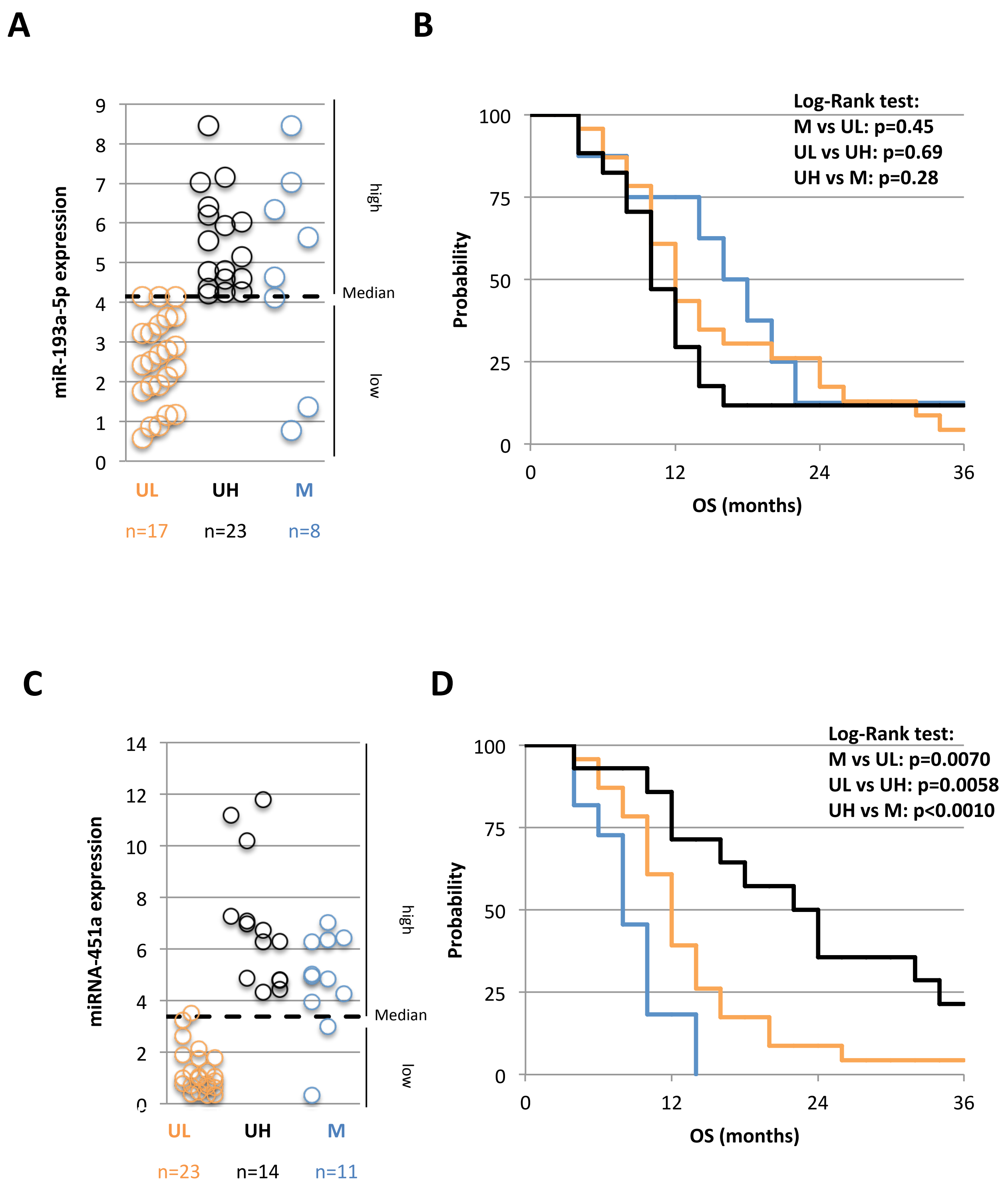
A
B
miR-193a-5p expression
9
8
7
6
5
4
3
2
1
0
high
Median
low
UL
UH
M
n=17
n=23
n=8
Log-Rank test:
M vs UL: p=0.45
UL vs UH: p=0.69
UH vs M: p=0.28
Probability
100
75
50
25
0
0
12
24
36
OS (months)
C
D
miRNA-451a expression
14
12
10
8
6
4
2
high
Median
low
UL
UH
M
n=23
n=14
n=11
Log-Rank test:
M vs UL: p=0.0070
UL vs UH: p=0.0058
UH vs M: p<0.0010
Probability
100
75
50
25
0
0
12
24
36
OS (months)

Figure S16.
