# Supplementary material for: Cytosine methylation of mature microRNAs inhibits their functions and is associated with poor prognosis in glioblastoma multiforme
Source: Mol Cancer. 2020 Feb 25;19:36. doi: 10.1186/s12943-020-01155-z (PMC7041276; doi:10.1186/s12943-020-01155-z)
Supplement: Supplementary file 8 — Additional file 8. Extended experimental procedures. [file 12943_2020_1155_MOESM8_ESM.docx]

**Cytosine methylation of mature microRNAs inhibits their functions and is associated with a poor prognosis in glioblastome multiforme.**

**Extended experimental procedures.**

1. ***Dot Blot.***

miRNA was treated in 0.1 M NaOH, at 95°C for 5 min, and then neutralized by adding 2μl of 6.6 M ammonium acetate. Next, 2-fold dilutions of denatured samples were spotted on a PVDF membrane in an assembled Bio-Dot apparatus (Bio-Rad, France). Vacuum was subsequently applied to filter through samples. The blotted membrane was washed with 2x SSC buffer, air-dried and vacuum-baked at 80°C for 2 hrs. The membrane was then blocked with 5% BSA/TBS-0,1%Tween and incubated with monoclonal 5-mC antibody (Active Motif, France). Binding of an HRP-conjugated secondary antibody was visualized by enhanced chemiluminescence. Image acquisition was performed on ChemiDoc MP (Bio-Rad, France).

1. ***Quantification of 5-methylcystosine by ELISA method.***

DNA was extracted using the QiaAmp DNA mini Kit (Qiagen, France) and miRNA extractions were performed by using the NucleoSpin® miRNA kit. The quantification of 5-methylcytosine is performed using the Methylamp Global DNA methylation Quantification kit (Euromedex-Epigentek, France) according to the manufacturer’s instruction.

1. ***HPLC-UV.***

The global miRNA methylation level was analyzed by HPLC according to Armstrong et al. (2011) and Bachère et al. (2017) with some modifications. miRNA sample (2μg) were digested with nuclease P1 (1.5 U/μg miRNA, Sigma , France) for 2 h at 37 °C, and then alkaline phosphatase (2.5 U/μg miRNA, Sigma France) was added for 1 h at 37 ◦C. After centrifugation (10 min, 7000×g, 4 °C), the supernatants were analyzed by HPLC coupled to UV detection (Agilent 1200 series). The nucleosides were separated at 10 °C on a Phenomenex Security Guard ULTRA Cartridges UHPLC C18 pre-column and a Phenomenex Kinetex 2.6 μm C18 100 A 100×4.6 mm column. The mobile phase was 50 mM diammonium hydrogen orthophosphate (diammonium hydro- genophosphate), 3% acetonitrile, pH 4.1 using the isocratic mode. The elution flow rate was set at 1.3 mL/min, and the pressure in the system was 330 bars. Commercially available nucleotides (dAMP, dTMP, dGMP, dCMP, dUMP) were injected individually and in a mixture (total injection volume of 13 μL) to determine their respective retention time following UV detection at 280 nm. Standard curves were also generated for dCMP (0.5–2.0 nmoles) and 5-mdCMP (0.015–0.100 nmoles) For the described conditions, the run time was 10 min and the retention times for both dCMP and 5-mdCMP were 1.4 and 2.2 min, respectively.

1. ***Western blot analyses.***

Proteins extracts were obtained by using RIPA Lysis and Extraction Buffer (Thermo Scientific, France) according to the manufacturer’s instructions.

Proteins were size fractionated by sodium dodecyl sulfate-polyacrylamide gel electrophoresis (SDS-PAGE) and transferred onto a nitrocellulose or PVDF membrane. Saturation and blotting were realized using the SNAP i.d™ Protein Detection System (Millipore, France). The detection of proteins was performed using ECL™(GE Healthcare, France) and/or SuperSignal west femto Maximum Sensitivity (Thermo Scientific, France) chemiluminscence reagents. Image acquisition was performed on ChemiDoc MP (Bio-Rad, France).

1. **Transient transfection of pre-miR^TM^.**

All the pre-miR^TM^ were obtained from Ambion and tranfected using the siPORT NeoFX Transfection Agent (Ambion, Life Technologies, France) according to the manufacture’s protocol. The pre-miR^TM^ Negative Control used is random sequences that have been tested in human cell lines and tissues and validated to not produce identifiable effects.

1. **Luciferase promoter and 3’UTR Reporter assay.**

Cells were seeded in 24-well plates and were transfected with the indicated firefly luciferase constructs together with an SV40-renilla control vector. Lysates were prepared at 40 hr, and luciferase activity was measured using the Dual Luciferase Reporter Assay system (Promega, Farnce) and a luminometer (MicroLumat Plus, EG&G Berthold, France).

1. ***Tumorigenicity assay and mice treatment.***

Cultured cells were harvested by trypsinization, washed and resuspended in saline buffer. Cell suspensions were injected s.c. in in the flank of 7-/8-week-old nude NMRI-nu female mice (Janvier, France).

Tumor volume based on caliper measurements were calculated by the modified ellipsoidal formula (*Tumor volume* = 1/2(*length* × *width*^2^)) according to previous data (Cartron et al., 2012). At the end of the 21-day observation period, the mice bearing xenograft tumors were killed and the tumor tissues were removed to be analyzed.

The experimental procedures using animals were in accordance with the guidelines of Institutional Animal Care and the French National Committee of Ethics. In addition, all experiments were conducted according to the Regulations for Animal Experimentation at the « Plate-forme Animalerie » of « Institut de Recherche en Santé de l'Université de Nantes (IRS-UN) » and approved by the French National Committee of Ethics (Aggrement number : B44278).

1. ***Patient characteristics.***

Overall survival was measured from the date of surgical resection to the death. In each tumor grade, all patients included in this study had similar management and similar treatment (including temozolomide (TMZ) for GBM).

Patient material as well as records (diagnosis, KPS, age, sex, date of death) was used with confidentiality according to French laws and recommendations of the French National Committee of Ethic. In addition, Patient material and experiments using this material are conducted according to the regulations of “the Réseau des thumarothèques du Canceropole Grand-Ouest” and more particularly with the regulations of “ Réseau Gliome”.

1. **Conditions of cell culture.**

U251 cells were routinely maintained in Dulbecco's modified Eagle's medium supplemented with 10% heat-inactivated fetal bovine serum, penicillin (10 IU/ml), streptomycin (10 μg/ml), and 10% non-essential amino acids (all purchased from Life Technologies, France).

1. ***Pull-down assay.***

Pull-down assays were performed using the His Tagged-Protein Interaction Pull-Down Kits (Thermo Scientific, France). Briefly, 100 μg of bait protein (His-DNMT3A, Methylation Ltd, USA) was immobilized on a column by incubation at 4 °C for 1 h with gentle mixing. After washing, 1μg of prey protein (GST-AGO4, ABNOVA) was added for 1 h at 4 °C with gentle rocking motion on a rotating platform. After washing and elution, the “bait-prey” interaction was analyzed by SDS-PAGE and Western blot.

1. ***Immunoprecipitation.***

Immunoprecipitations were realized by using the Catch and Release® v2.0 Reversible Immunoprecipitation System (Millipore, France) with 4 μg of antibody of interest.

1. ***Subcellular fractionation.***

Nuclear extracts were prepared by using the Nuclear Complex Co-IP Kit (Active Motif, France). Subcelllular fractions were obtained using the Nuclear Extract Kit (Active Motif, France).

1. **Synthetic methylated miRNA.**

Synthetic methylated and unmethylated miRNAs are provided by Sigma (France).

1. **Cell Proliferation.**

Cell counts and viability were performed using the Countess optics and image automated cell counter (Life Technologies). Cells were mixed with Trypan blue (50/50) and loaded into a Countess chamber slide. The image analysis software was used to automatically analyze the acquired cell images from the sample to give cell count and viability. Data were plotted as the number of viable cells for cell proliferation or compared with proliferation in control condition for relative proliferation.

1. **Invasion assay.**

All of the procedures were performed according to the manufacturer’s instructions (QCM 24-Well Collagen-Based Cell Invasion Assay, Millipore, France). In brief, 200 μl of serum-free medium containing 2 × 105 cells were added into the invasion chamber with the bottom well of the 24-well plate containing 500 μl of complete medium. After 72h of incubation at 37°C, the medium was removed and the cells were stained by placing the chamber in staining solution for 20 min at room temperature. Cells that did not invade were carefully removed from the top-side of the chamber using a cotton swab. The stained chamber was inserted into a clean well containing 200 μl of extraction buffer for 15 min at room temperature. A total of 100 μl of extracted (stained) solution from the chamber was transferred into a 96-well plate and the optical density was measured 570 nm using a spectrophotometer.

1. ***In-Cell ELISA.***

In cell ELISA were performed using the *In-Cell* ELISA Kit (Abcam, France) according to the manufacturer’s instructions, and after a fixation step performed with 4% of paraformaldehyde solution (10 min at room temperature). Primary antibodies were incubated overnight at 4°C. Adequate HRP-conjugated secondary antibodies were incubated for 1 hour at room temperature. Detection was performed at 450 nm.

After the washes, cells in each well were incubated with 1X Janus Green Stain for 5 min at room temperature, according to the manufacturer’s instructions. Data were expressed in normalized unit, according to the following calculation: (HRPsignal ‘minus’ HRPsignal in absence of primary antibody)/(Janus Green signal ‘minus’ Janus Green signal in absence of cells).

1. **List of antibodies.**

| ***Antibody*** | ***Reference*** |
| --- | --- |
| 5mC | Abcam#Ab10805 and Active Motif#39649 |
| m6A | Active Motif#61755 and Abcam#208577 |
| AGO1 | Abcam#Ab129304 and Santa Cruz#sc-32657 |
| AGO2 | Abcam#57113 and Santa Cruz#sc-53521 |
| AGO3 | Abcam#3593 |
| AGO4 | Active Motif#39855 Abcam#85077 et Santa Cruz#sc-374220 |
| NSUN2 | Santa Cruz#sc-366094 and Abcam#128243 |
| DNMT2 | Santa Cruz #sc-20702 and |
| DNMT1 | Active Motif#39204 and Santa Cruz#sc-10221 |
| DNMT3A | Active motif #39206, and Abcam#2850 and Santa Cruz#sc-20703 |
| DNMT3B | Active Motif #39207 and Santa Cruz#sc-20704 |
| IgG | Santa Cruz#sc-66931 and Abcam#Ab37373 |
| TP73 | Santa Cruz#sc-17823 |
| β-Actin | Santa Cruz#sc-81178 |
| Dicer | Active Motif#39817 and Abcam#1282434732 |
